# Genomic alterations in gastric cancers discovered via whole-exome sequencing

**DOI:** 10.1186/s12885-018-5097-8

**Published:** 2018-12-19

**Authors:** Jie Zhang, Weiqing Qiu, Hua Liu, Changlin Qian, Dujuan Liu, Hailong Wang, Ni Hu, Y. Tom Tang, Jianhua Sun, Zhiyong Shen

**Affiliations:** 1 Department of General Surgery, South Campus, Renji Hospital, School of Medicine, Shanghai Jiao Tong University, 2000 Jiangyue Road, Shanghai, 201112 China; 2 First Dimension Biosciences (SuZhou) Co., Ltd, Building B8, Floor 5, BioBay, 218 Xinghu Street, Industrial Park District, Suzhou, 215126 Jiangsu China

**Keywords:** Whole-exome sequencing, Candidate mutations, Gastric adenocarcinoma (GAC), SNP, Metastasis, Copy number variation (CNV)

## Abstract

**Background:**

Gastric cancer (GC) ranks the second in mortality rate among all cancers. Metastases account for most of the deaths in GC patients. Yet our understanding of GC and its metastasis mechanism is still very limited.

**Methods:**

We performed 20 whole-exome sequencing (WES) on 5 typical metastatic gastric adenocarcinoma (GAC) patients with lymph node metastasis. We compared both the primary tumors to their metastatic lymph nodes, and a specific analysis pipeline was used to detect single nucleotide variants (SNVs), small insertions/deletions (indels) and copy number variants (CNVs).

**Results:**

(1) We confirmed 30 candidate mutations in both primary and lymph nodes tissues, and other 7 only in primary tumors. (2) Copy number gains were observed in a large section of 17q12–21, as well as copy number losses in regions containing *CDKN2A* and *CDKN2B* in both primary and lymph nodes tissues.

**Conclusions:**

Our results provide preliminary insights in the molecular mechanisms of GC initiation, development, and metastatic progression. These results need to be validated through large-scale studies.

**Electronic supplementary material:**

The online version of this article (10.1186/s12885-018-5097-8) contains supplementary material, which is available to authorized users.

## Background

Gastric Cancer (GC) is one of the most common cancers in the world, coming fourth and second in incidence and mortality rates, respectively [1]. Most GCs are gastric adenocarcinomas (GACs), which further progress into intestinal and diffuse types based on the Lauren classification [2]. Statistical data indicate that about 52% of the world’s GAC patients come from China [3]. Although the incidence and mortality rates of GC are decreasing, there are still around 700 thousand reported new cases and 500 thousand deaths per year [4]. The high mortality rate is largely due to the lack of efficient early diagnosis, tumor metastasis in the advanced stage, and a lack of GC-specific precision-class medications. Therefore, it remains an urgent task to study its molecular mechanism of tumorigenesis and metastasis, so as to promote familial genetic screening, early diagnosis, and the development of effective therapeutic agents based on cancer-specific mutations and biomarkers.

GC is closely associated with susceptible genetic variants [5, 6]. The main reason for the development of GC is the activation of proto-oncogene and the inactivation of tumor suppressor genes [7].

In recent years, we have improved understanding of the molecular aspects of GC. In particular, next-generation sequencing (NGS) provides a high-throughput method to systematically identify genetic alterations in the cancer genome. Several NGS studies in GC have been conducted, and many driver gene mutations reported, including *TP53*, *PIK3CA*, *CTNNB1*, *CDH1*, *SMAD4 and KRAS* [8]. Some tumor suppressor genes, such as *APC*, *CDH1*, *CDH4*, *THBS1* and *UCHL1* are found to be inactivated by hypermethylation [9–12]. About 59% of GCs have mutations in chromatin remodeling genes such as *ARID1A*, *PBRM1 and SETD2* [13]. Wang’s study also found new mutated driver genes (*MUC6*, *CTNN2A* and *GLI3*) through whole-genome sequencing [14]*.* Furthermore, they found that 14% of diffuse-type tumors have *RHOA* gene mutation [14]. *CDH1* mutations were reported in hereditary diffuse GC [15].

Today, genomic level characterization of GC is still lacking, in comparison to lung or colon cancers. We do not have a conclusive grasp on genomic level alterations, possibly due to its lower occurrence rate in developed countries. There are even fewer genomic level studies on the mechanism of GAC metastasis.

## Methods

### Patients and specimen collection

Five patients, all ethnic Chinese Han people, were selected from our GAC patient pool (over 1000 patients annually in Renji Hospital, Shanghai, China) based on sample completeness and quality. All patients selected were at an advanced disease stage at initial presentation (stage III or IV in AJCC 7th staging system) and had received curative or palliative gastrectomies. These patients did not receive preoperative chemotherapy or radiotherapy before surgery. Table 1 contains basic information concerning these patients. For each patient, we collected the primary gastric cancer tissue, the adjacent stomach normal tissue, the metastatic lymph nodes and the adjacent normal lymph nodes. All tumor samples were confirmed to have at least 20% tumor cells in proportion, while adjacent normal samples have 0% tumor cells in proportion through H&E staining.

**Table 1:** Overview of patients and tumor characteristics of the 5 GAC analyzed in the study

ID	Gender	TNM	Stage	Histological type
P1	Male	T4bN3aM0	IIIC	Mucinous adenocarcinoma
P2	Male	T4bN3aM0	IIIC	Papillary tubular adenocarcinoma
P3	Male	T4bN3aM0	IIIC	Undifferentiated adenocarcinoma
P4	Male	T4bN3bM1	IV	Poorly differentiated
P5	Male	T4bN3bM0	IIIC	Mucinous adenocarcinoma; Signet ring cell

### Nucleic acid preparation

Tissue sections were deparaffinized, as previously reported, by using 100% xylene (Sigma Chemical Company, St. Louis, Mo), followed with 100% ethanol [16]. The deparaffinized samples were then suspended again in one proteinase K-contained buffer (Finnzyme, Espoo, Finland). After extracted with phenol-chloroform (Sigma Chemical Company), the DNA samples were treated with ethanol precipitation and resuspension in deionized water. DNA was quantified through spectrophotometer, and 200 ng DNA samples were utilized as a template for each polymerase chain reaction (PCR).

### Whole-exome sequencing and data analysis

Library construction and targeted exome enrichment were performed using the Illumina TruSeq DNA Sample Prep Kit (San Diego, CA, USA) and the SeqCap EZ Human Exome Library v2.0 kit (Roche NimbleGen, WI, USA), respectively. Next, paired-end sequencing was performed on the Illumina X-Ten sequencer, according to the manufacturer’s instructions, yielding ~ 150 bp short sequence reads. For each sample, there were around 100 million reads generated, accounting for 100-200X coverage of the entire exome.

Raw reads (fastq files) were checked for the data quality using Fastqc [17]. Plots of quality scores across all bases in reads showed the majority of positions have quality Q ≥ 20. Raw reads were then trimmed for adapter contamination with Trimmomatic version 0.32 [18]. Leading and trailing low quality bases (below 3) were removed. Reads were also scanned with a 4-base wide sliding window and the following bases were cut when the average quality per base drops below 15. Finally, only reads longer than 50 bases were kept for next step analysis. Paired clean reads, after Trimmomatic treatment, were aligned against the reference genome hg19 by using Burrows-Wheelers Aligner [19]. The remaining reads were then calibrated and realigned using Genome Analysis Toolkit [20]. The realigned BAM files were analyzed using MuTect [21] to detect somatic single-nucleotide variants and insertions/deletions, respectively. Normal germline variants were filtered out by dbSNP database (dbSNP version 132) [22]. All programs were run under default parameter settings. Copy number variation analysis was performed by EXCAVATOR2 [23]. A copy number (CN) of 2 meant no CNV i.e. the cancer tissue having the same CN as the healthy control. CN > 2 means a copy gain in some paired chromosomes, and CN < 2 means the loss of at least one copy within the chromosome pair.

### Confirmation of germline and non-synonymous somatic mutations

We exploited the Verity 96-well PCR amplifier (ABI, USA) to perform PCR by adding special primers, followed by conventional PCR-based Sanger sequencing using the ABI3730XL (ABI, USA) sequencer. Next, we compared the results with the next-generation sequencing data to confirm the germline and nonsynonymous somatic mutations. For some low VAFs below detection limit of Sanger sequencing, we use IGV (Integrative Genomics Viewer) software [24] to check the results manually. The mutations with both base count more than 10% and QV (Quality value) more than 20 were considered to be trusted mutations.

## Results

### Identification of somatic variations from primary tumor and lymph node metastatic tissues

**Fig. 1:**
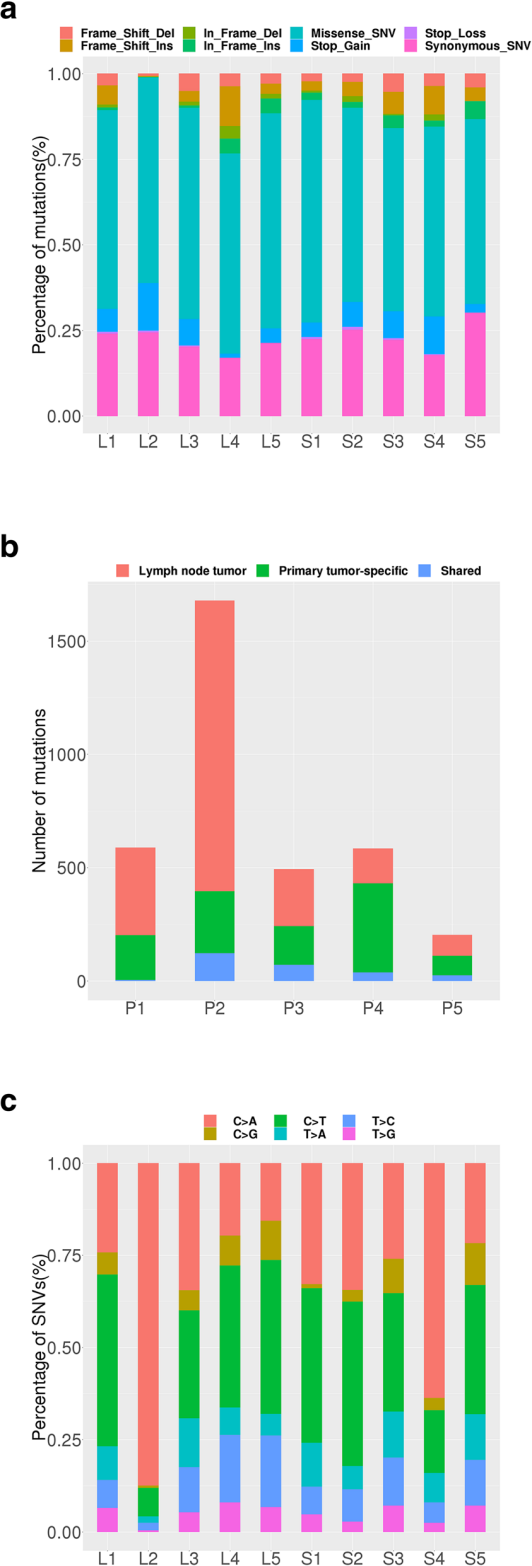
Statistical analyses of somatic mutations in 5 GAC patients. **a** Somatic mutation types and their frequency in 5 GAC patients. S1~S5: primary tumor tissue; L1~L5: lymph node metastatic tissue; 1~ 5: patient ID. Seven colors represent seven kinds of mutations, as shown inside the figure. **b** Somatic mutation number in 5 GAC patients. P1~P5: patient ID. Red means lymph node metastatic tissue specific; green means primary tumor tissue specific; blue means both. **c** The proportion of base substitutions of somatic mutations in 5 GAC patients. S1~S5: primary tumor tissue; L1~L5: lymph node metastatic tissue; 1~ 5: patient ID. Six color means six kinds of mutations

Average sequencing depth of the protein-coding regions were ~ 244.6X in primary tumors, ~ 224.2X in normal stomach tissues, ~ 220.8X in normal lymph nodes, ~ 230.8X in metastatic lymph nodes (see Additional file 1: Table S1 for detailed info). We detected a large collection of SNPs and indels in both primary and lymph node metastatic tissues for all the five GAC patients. Overall, 3228 translational meaningful somatic mutations were detected. The main type of mutation is nonsynonymous SNV (61.6%). Figure 1a shows the mutation types and frequency that were detected. All mutations reported here are within the protein coding regions, and we also excluded these variations with greater than 5% frequency as reported in either the dbSNP database [22] or the ExAC database [25]. By comparing with the normal tissues, common and unique gene mutations were selected from different patients and tissues. The number of mutations in primary tumors and lymph node metastatic tissues are shown in Fig. 1b. From Fig. 1b, we can see P2 has the highest number of SNVs; 274 in primary tumor tissue only, 1281 in lymph node metastatic tissue only, and an additional 123 SNVs common to both tissues. Complete list of all SNPs was provided in Additional file 2: Table S2.

To dissect a mutational signature of primary tumors and metastatic lymph nodes, we examined the spectrum of base substitutions. C-to-A was the most frequent transversion in somatic mutations, which account for ~ 14.9% and ~ 44.3% of somatic mutations in primary tumors and metastatic lymph nodes, respectively (Fig. 1c. C-to-T transversion was also enriched in the somatic mutations, consistent with previous studies [26, 27].

### Biological processes implicated

**Fig. 2:**
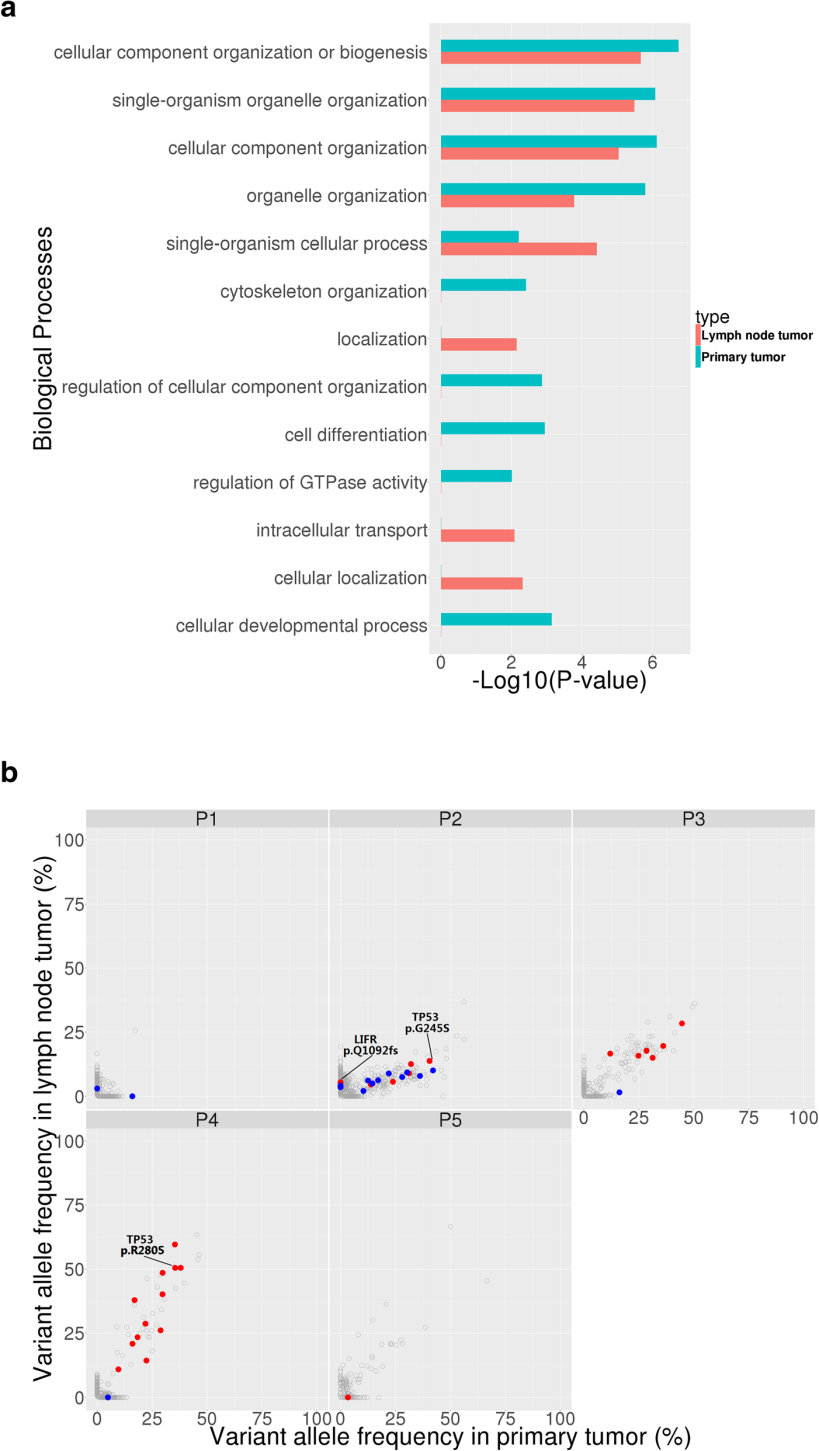
The distribution of somatic mutations between primary tumors and lymph node metastatic tissues. **a** Biological processes frequently mutated in primary tumors and lymph node metastatic tissues. The functional terms significantly overrepresented are presented as -log_10_ (*P*-value). Red means lymph node metastatic tissue; blue means primary tumor tissue. **b** Paired variant allele frequency of mutations in primary tumors and lymph node metastatic tissues. Gray point: mutations with VAF ≥ 5% in primary and lymph node metastatic tissues; Blue point: mutations with VAF ≥ 10% in primary tumors; Red point: common mutations with VAF ≥ 10% between primary and metastatic tissues; P1-P5: Patient ID; VAF:Variant allele frequency. *TP53* is identified as a common driver genes, since they occur in multiple patients as both somatic and germline mutations. They are further proposed to be novel drug targets for cancer therapies, with further target validation required

We examined biological processes with frequently mutated genes in primary tumors and metastatic lymph nodes. Consistent with a previous report [28], genes involved in cell adhesion and chromosome organization were frequently mutated in our GAC patients. Cellular component organization or biogenesis was the most enriched biological pathways among the frequently mutated genes within both primary tumors and lymph node metastatic tissues. Besides, single-organism organelle organization, cellular component organization, organelle organization and single-organism cellular process are the common pathways. Clustering of these mutations reveals many unique pathways as well. For instance, in primary tumors there are cytoskeleton organization, regulation of cellular component organization, regulation of GTPase activity, cell differentiation and cellular developmental process pathways; in metastatic lymph nodes there are localization, intracellular transport and cellular localization pathways (Fig. 2a).

### Confirmation of somatic non-synonymous mutations

In order to discover new driver gene candidates, we focus on the mutations (VAF ≥ 10%) from more than 3000 cancer-related genes reported in the Cancer-genetics web [29]. This collection of genes is speculated to be cancer related and potentially cancer driving. We compared the SNPs from both primary and metastatic tissues of all patients and found 37 such mutations within 35 genes from 5 patients.

These mutations were then successfully confirmed by Sanger sequencing and IGV (Integrative Genomics Viewer) software [24] (for detail, please see Additional file 3: Figure S1). Figure 2b marked the variations of these genes within the primary and metastatic tissues. The number of genes mutated varies greatly among 5 patients, with P2 having the most (16 mutations), and P5 the least (1 mutation). (Additional file 4: Table S3 shows all the somatic mutations identified in primary tumor and metastasis tissues from 5 patients by Sanger sequencing and IGV).

In particular, 30 somatic SNVs were confirmed in 28 genes (*TRIM24, LIFR, EPHA3, FOXG1, ABCC4, PTEN, SOX5, AKAP13, NTRK3, KLK10, FBXW7, ABCB1, LRP6, ARID1A, LIG3, TP53, ACTN4, LEF1, TLR2, PKHD1, CSMD1, CYP24A1, BCR, ITGAX, PTK7, SLCO1B1, JUN, CHCHD7*) for both the primary tumors and lymph node metastatic tissues, and 7 mutations unique to the primary tumor tissues. Among the seven unique mutations, 4 of them have been reported once whereas the other three haven’t been recorded in the COSMIC database at all [30]. These 7 mutations were mainly carried by three patients, of which Patient 2 carried the most mutations (See Table 2 for detailed info about these 7 mutations). Approximately 72% of somatic mutations were shared between the primary and metastatic sites. By comparing our findings to 578 known driver genes from the COSMIC database [30], we found that among the 35 genes, 12 of them are known cancer driver genes (*EPHA3, TRIM24, BRAF, LIFR, PTEN, NTRK3, FBXW7, ARID1A, TP53, JUN, CHCHD7, SMAD4*).

**Table 2:** The unique mutations in the primary tumor tissue

Sample ID	Gene	Mutation	NM ID	Base changes	AA changes	Domain	VAF	N in Cosmic**(**tissue**)**
P2_S	*BRAF*	frameshift insertion	NM_004333	c.1208dupC	p.P403fs	–	0.26	1 (Endometrioid carcinoma)
P2_S	*CD276*	nonsynonymous SNV	NM_001024736	c.C479T	p.T160 M	CD80-like_ immunoglobulin C2-set	0.149	–
P2_S	*CUL3*	nonsynonymous SNV	NM_003590	c.G1091A	p.R364H	Cullin repeat-like-containing domain	0.102	1 (Kidney)
P2_S	*EPHA7*	nonsynonymous SNV	NM_004440	c.T1056G	p.S352R	Fibronectin type III	0.11	–
P2_S	*NKX2–2*	nonsynonymous SNV	NM_002509	c.C413T	p.A138V	Homeobox domain	0.163	1 (Stomach)
P3_S	*SMAD4*	nonsynonymous SNV	NM_005359	c.G1082C	p.R361P	SMAD domain_ Dwarfin-type	0.166	1 (Stomach)
P1_S	*STAT6*	nonsynonymous SNV	NM_003153	c.C1210A	p.Q404K	STAT transcription factor_ DNA-binding	0.118	–

*P1~P5* patient ID, *VAF* Variant Allele Frequency, *AA* amino acid, *N* in Cosmic(tissue), The results come from Cosmic website https://cancer.sanger.ac.uk/cosmic

### Copy number variations in primary tumor and lymph node metastatic tissues

**Fig. 3:**
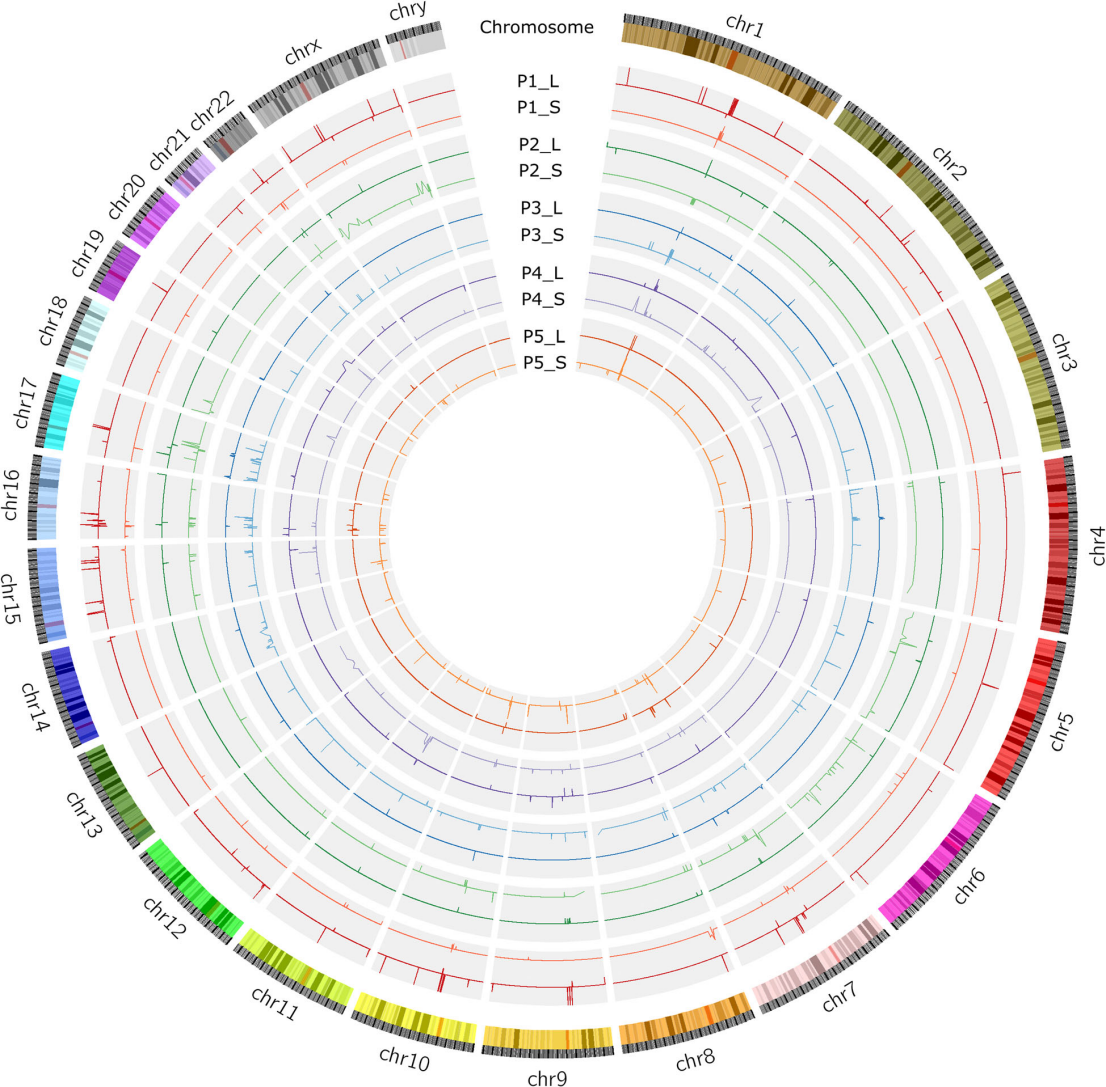
View of CNV aberrations across each chromosome for 5 patients. Sample IDs are composed of Patient ID, followed by “-”, followed by a letter. P1~P5 are Patient IDs; letter L indicates lymph node metastatic tissue; and S primary tumor tissue. CNVs are depicted as spikes (ups and downs) within the circular curve. Shared CNVs among different cancer tissues are seen as similar spikes in the same angular region from the circle center. Within the figure, we can visually detect multiple CNVs among multiple samples

It has been established that copy number variation (CNV) arises as a result of preferential selection that favors cancer development [31]. Figure 3 shows the overall CNV situations among all 10 cancer samples from 5 patients. The outmost circle shows chromosomal positions. The next one shows gene distributions. As we are only sequencing exomes, the intergenic regions are not captured within this figure. The 5 interior circles represent 5 patients, each showing one primary cancer tissue, and one lymph node with metastatic cancer. CNV levels were obtained by dividing the two numbers: the total gene segment sequence counts seen in each gene in either the cancer tissue or the adjacent normal tissue. To address the concern of CNV variations among the normal tissues, we did compare the adjacent normal stomach tissue to the adjacent normal lymph nodes, and as expected, we did not detect any significant CNVs.

Our CNV graph resolution is at gene level. After carefully examine this graph, we discovered a total of 4130 genes with some sort of CNVs among the 10 samples. In the primary cancer tissues, CN gains in 3260 genes, and losses in 618 genes. In metastatic lymph nodes, CN gains in 834 genes and losses in 182 genes. So, the primary cancer cells have more CN gains than that of metastatic cells. Among the 5 patients, P3 has the most CNV events and P1 has the least.

Table 3 shows more detail about the CNVs, including quantities of those found in each specific tissue or those found shared between multiple tissues. The CN gains specific to the primary tumors total 2601 genes, while there are 332 CN losses, clustered in chromosome 4q and 9p. There were only 580 CN gains in the metastatic lymph nodes and CN losses were observed in 47 genes, clustered in 1q and 8p. The shared CNVs in both metastatic lymph nodes and primary tumors number 332 genes. (Details provided in Additional file 5: Table S4).

**Table 3:** Summary of CNV types and numbers in primary tumor and metastatic samples of 5 patients

ID		Num (S-Gain)	Num (S-Loss)	Num (L-Gain)	Num (L-Loss)
P1		113	39	61	4
P2		791	446	9	70
P3		1241	19	238	1
P4		1093	5	395	25
P5		22	109	131	82
Total		3260	618	834	182
Status		Num	Main locus		CN
Unique	S-Gain	2601	1p12; 1p13; 1q21; 1q23; 1q32; 1q42; 11q13; 13q12; 13q11; 13q22; 13q32; 13q33; 14q21; 14q22; 5p12;17p13; 17q21; 5p13; 5p15; 6p21; 6p22; 7q11; Xp22		2~ 16.67
	S-Loss	332	4q32; 4q33; 4q35; 9p21; 9p22; 9p23; 9p24; 11p15; 16p13; 18p11		0.52~ 2
	L-Gain	580	1p11; 1q21; 10p12; 10q11; 11q13; 12p13; 14q32; 15q13; 16p13; 17q11; 20q11; 20q11; 20q13		2~ 7.04
	L-Loss	47	1q21; 10q11; 16p13; 2p24; 22q11; 8p23		0.41~ 2
Common	S-Gain L-Gain	279	1q21; 11q13; 14q24; 17p13; 17q12; 17q21; 17q24; 19p13; ap23; 21q22; 4q12; 7p22; 7q11; 8p23; 9p12; 9q12; 9p13; 9q31;		2.00~ 7.04
	S-Loss L-Loss	52	1q21; 10q11; 11p11; 11q12; 15q24; 16p13; 16p12; 17q12; 2q13; 14q11; 22q11; 5q13; 1p36; 5q35; 6p21; 8p23; 9p21; 9p12; 9p11.2		0.11~ 1.42
	S-Loss L-Gain	1	7q22.1		0.91~ 1.12 3.42~ 7.04

*P1~P5* patient ID, *S* primary tumor tissue, *L* lymph node metastatic tissue, *CNV* copy number variation, *Num* gene numbers, *S-Gain* CNV gains of primary tumor tissues. S-Loss, CNV losses of primary tumor tissues. L-Gain, CNV gains of lymph node metastatic tissues. L-Loss, CNV losses of lymph node metastatic tissues. *CN* copy number

To further understand tumorigenesis in the primary tissues and in metastasis process, we carefully examined the 578 cancer driver genes from the COSMIC database [30]. Table 4 shows the CNVs either unique or common in the primary and metastatic tissues. Our data confirms CNVs reported in other GC studies, including important driver genes such as *EGFR*, *ERBB2*, *ERBB4*, and *CCND1* [32, 33]. *EGFR* copy number gains were associate with an increased risk of invasion and metastasis in solid tumors including GC, suggesting its potential significance as a prognostic marker [31]. It is interesting that *EGFR* has a large gain in patient P2 in both the primary tissue (CN = 4.54, compared to the adjacent normal tissue), and in the metastatic tissue (CN = 19.95). In patient P5, however, there is a CN loss (CN = 0.48) in its lymph node metastatic cells.

**Table 4:** A detailed overview of DNA copy number gains or losses in primary tumors and metastatic tissues

Unique to the primary tumor tissues							
Locating Gene	Locus	V	CN	Locating Gene	Locus	V	CN
* ACSL3, PAX3*	2q36.1	+	3.49	*MAD2L1BP*	6p21.1	+	2.69
* BRCA1*	17q21.31	+	3.79	*MSH6, FBXO11*	2p16.3	+	3.21
* BRCA2, KL*	13q13.1	+	2.66	*MTCP1*	Xq28	+	3.62
* CCNB1IP1*	14q11.2	*+*	3.31	*MTUS2, FLT1*	13q12.3	+	2.66
* CCND1*	11q13.3	*+*	5.83	*MYB*	6q23.3	+	6.07
* CDK12*	17q12	*+*	6.5	*NCOA4*	10q11.23	+	2.93
* CDK8*	13q12.13	+	2.66	*NIN*	14q22.1	*+*	3.44
* CDX2, FLT3*	13q12.2	+	2.66	*NOTCH2*	1p11.2	+	4.68
* EGFR*	7p11.2	+	4.54	*NRAS, TRIM33*	1p13.2	+	4.68
* ELK4, IKBKE, SLC45A3*	1q32.1	+	2.74	*PDE4DIP*	1q21.1	+	7.66
* EPCAM, MSH2*	2p21	+	3.21	*RABEP1, USP6*	17p13.2	+	2.82
* ERBB2*	17q12	+	3.76~ 6.5	*RARA, TOP2*	17q21.2	+	3.76~ 6.5
* ERBB4, IDH1*	2q34	+	2.86	*RB1*	13q14.2	+	2.64
* ERCC5*	13q33.1	+	2.64	*SMAD2*	18q21.1	+	3.53
* FAM46C, HSD3B2*	1p12	+	4.68	*STAT3*	17q21.2	+	3.79
* FEV, ATIC*	2q35	+	2.08~ 3.49	*SUZ12*	17q11.2	+	2.71
* FGFR3*	4p16.3	+	4.21	*TERT*	5p15.33	+	3.03
* KRT17*	17q21.2	+	3.08	*TRIM27*	6p22.1	+	2.97
* KTN1*	14q22.3	*+*	3.44	*YWHAE*	17p13.3	*+*	4.21
* LCP1*	13q14.13	+	2.64	*ZMYM2*	13q12.11	+	2.66
* LRP5*	11q13.2	+	5.83	*ZRSR2*	Xp22.2	+	3.23
Unique to the metastatic tissues							
Locating Gene	Locus	V	CN	Locating Gene	Locus	V	CN
*CHD6, MAFB, TOP1*	20q12	+	3.05	*SRC*	20q11.23	+	3.05
*NUMA1*	11q13.4	+	2.94				
Common to the primary tumor and metastasis tissues							
Locating Gene	Locus	V	CN	Locating Gene	Locus	V	CN
*HIP1*	7q11.23	+	2.65~ 2.77	*RAD51B*	14q24.1	*+*	2.75–2.78
*CHIC2, KDR, KIT, PDGFRA*	4q12	+	2.67~ 3.17	*ETV4*	17q21.31	+	2.66–5.11
*TMPRSS2*	21q22.3	+	2.90~ 3.88	*CDKN2A*	9p21.3	–	0.8–1.32
*PMS2*	7p22.1	+	2.62~ 2.78	*CDKN2B*	9p21.3	–	0.8–1.32

*V* CNV state, *CN* Copy number, +, CNV gain; —, CNV loss

**Fig. 4:**
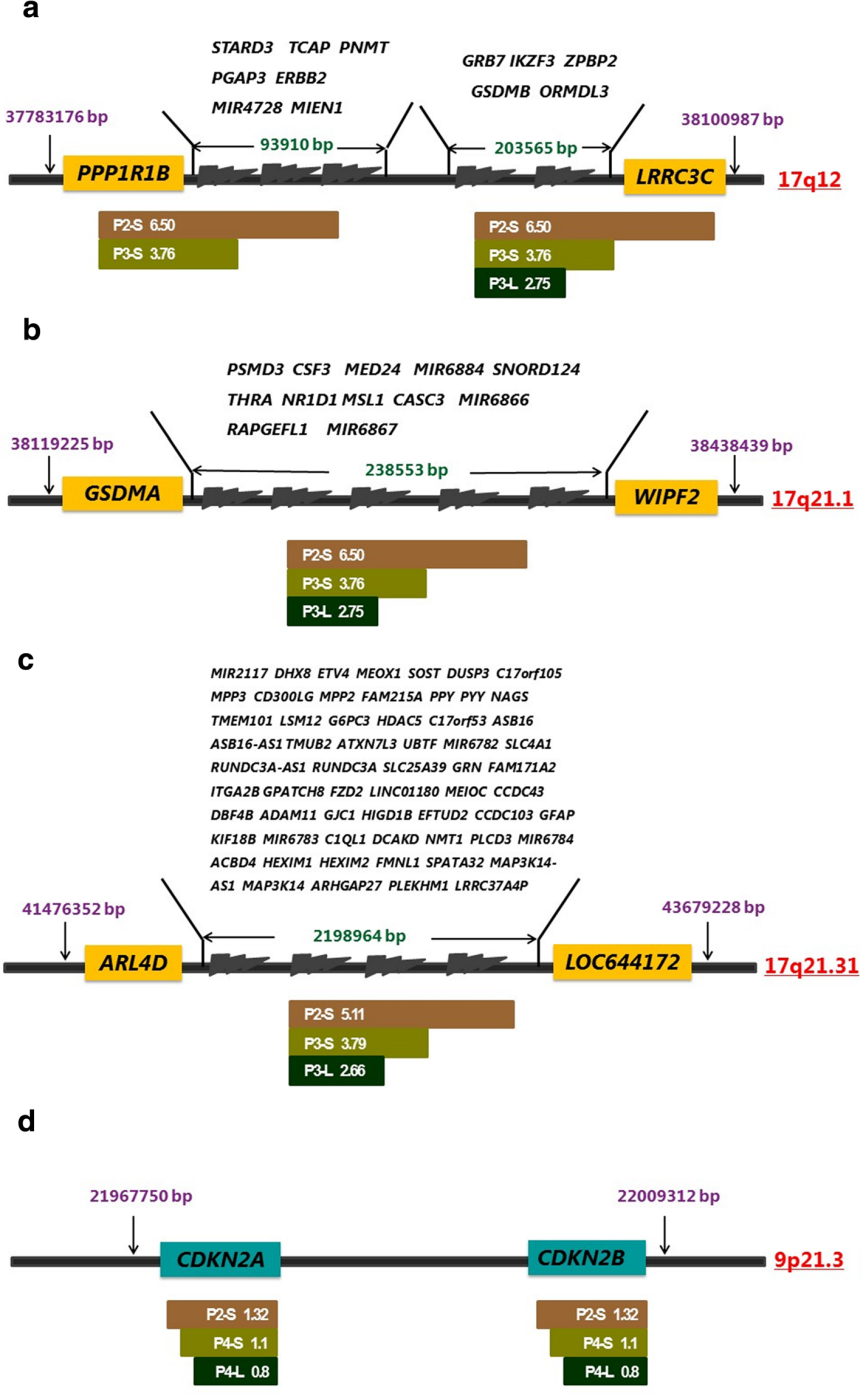
CNVs in different samples with boundary resolution at gene level. CNV gains and losses involving key driver genes in certain samples are shown here. Genome sequences are represented by the central axis, with genomic arm locations given on the right side. Their genomic starting and ending base pair locations were indicated in pink numbers ending with bp. Since our results are achieved through exome sequencing, the boundaries demarked here represent the contained regions within the real CNV blocks instead of the exact basepair locations. Gene IDs within the CNV blocks are either in yellow (**a**-**c**) or green (**d**) boxes, or listed above the central axis. Sample IDs are listed below the central axis, with their respective CN listed on the right side, in white. A CN greater than 2 is interpreted as CNV gains and a CN less than 2 as CNV losses. Sample IDs start with Patient IDs (P1-P5), followed bya hyphen and tissue type (S or L). S represents primary cancer tissue, and L metastatic lymph sites. **a** Likely two blocks of CNV gains involving *ERBB2*. One block starts with *PP1R1B* and ends with *LRRC3C*, with a genomic length of ~ 318Kb containing a total of 14 genes. Tissues include P2-L and P3-S. The other one, in P3-L, starts at *GRB7* and ends after*LRRC3C*. **b** Genomic block amplification in 17q21.1, starting at *GSDMA* and ending at gene *WIPF2*. The block size is ~ 319Kb, involving 4 tissues (P2-S, P2-L, P3-S, P3-L). This amplification is likely critical for both tumorigenesis and metastatics, as the amplification is present in both primary tumors and metastatic tissues with the exact same boundaries. CN is from 2.75 to 6.50. **c** Another block with CNV gains in 17q21.31. This block is the largest, with genomic length of 2,202Kb, and involving 63 genes. The boundaries are most likely fixed in three samples (P2-S, P3-S, P3-L). **d** A CNV loss event in 3 samples (P2-S, P4-S, P4-L) in chromosome 9p21.3, involving two cancer driver genes (*CDKN2A* and *CDKN2B*). The block size is 41Kb, a relatively small block. It is likely that one copy of these two genes was lost in one of the chromosomes. The differences in the CN in different tissues are small, and could simply be caused by the portion of cancerous cells within the sample tissues. This loss may be important to tumorigenesis and metastasis

The chromosome 17q arm is particularly plagued by CNVs and warrants a closer look. Figure 4a-c gives the genomic regions where CN gains occur. In Fig. 4a, we see an important gene, *ERBB2* has CN gains in 3 of the 5 patients, ranging 3.76~ 6.5. Additionally, all the neighboring genes within the same location (17q12) show similar CNV pattern in a total of 3 samples involving 5 patients. The block of genes amplified multiple times, starting from the left of *PPP1R1B* (17q12, location 37,783,176), and ending to the right of *LRRC3C* (17q12, location 38,100,987), a segment of ~ 318 KB involving 14 genes.

There are other segmental CNV gains in 17q21.1 and 17q21.31, involving 14 and 63 genes respectively, with CN at 2.66~ 5.11, and each in at least 3 distinct tissues. The average amplification folds for each segment in each positive sample are listed in Fig. 4b, c. Although there were several reports previously concerning 17q segmental amplifications, nobody has provided exact boundaries of these segments [32–34]. Through exome sequencing, we are the first to provide exact boundaries of these segments with resolution at the gene level. The duplication events most likely happened at two exact positions (hotspots) with the genome, and it happened multiple times. Unfortunately we did not do whole-genome sequencing, cannot identify these two hotspots. They can be resolved by whole genome sequencing.

We also detect another interesting CN loss in two known tumor suppressor genes, *CDKN2A* and *CDKN2B*, with CN at 0.8~ 1.32, as shown in Fig. 4d. It occurred in 2 patients, involving 3 samples (primary tumors and metastatic tumors). The segment is about 32 KB in 9p21.3. This CN < 2 is likely the result of losing one copy of the gene in majority of cancerous cells, as the CN is close to 1.0. However, we cannot rule out the possible loss of both copies in 2 chromosomes in a smaller portion of the sequenced cells. Since the expression level of *CDKN2A* and *CDKN2B* are critical to tumorigenesis, we believe the CN losses of these two genes contribute to the malignancy of the tumor.

## Discussions

We found that genes involved in cell adhesion and chromosome organization were frequently mutated in these GAC patients, which is consistent with previous reporting [23]. There are many different somatic mutations between primary tumor tissues and their paired lymph node metastatic tissues, especially in Patient 2. We confirmed that 30 candidate driver mutations were mutated in both primary and lymph nodes tissues, and seven candidate mutations which only mutated in primary tumors. Theoretically it is easy to understand that there are more mutations within the primary tumors than that of the metastatic tumors, as the metastatic tumors comes from a subpopulation of the primary tumor. It is somewhat surprising that we did not see any metastatic specific mutations, as one would expect these metastatic tumor cells will continue to mutate its genome and to gain more growth power. Also, we found that there were no common mutations among the five patients, suggesting a high heterogeneity of tumor cells and the complexity of the pathogenesis in gastric tumor. It will require a much larger population study to outline the possible subtypes of GAC based on driver mutation collections.

For CNVs, although there were several reports regarding 17q segmental amplifications, nobody has provided exact boundaries of these amplified segments. It has been reported that the *PPP1R1B-STARD3-TCAP-PNMT-PERLD1-ERBB2-MGC14832-GRB7* locus at chromosome 17ql2 is frequently amplified in gastric cancer and breast cancer [32]. Maqani et al. discovered that the large section sequence amplification is closely related to GC incidence [33]. Varis et al. used GC xenografts and four GC cell lines to confirm that 11 genes within 17q12–21 region were amplified within the genome (*ERBB2*, *TOP2A*, *GRB7*, *ACLY*, *PIP5K2B*, *MPRL45*, *MKP-L*, *LHX1*, *MLN51*, *MLN64*, and *RPL27*) [34]. Through exome sequencing, we are the first to provide exact boundary resolution of these segments at the gene level. The duplication events most likely happened at two exact positions (hotspots) within the genome, for a fixed chunk of the chromosome.

In addition, *CDKN2A* and *CDKN2B, at chromosome 9p21 and encoding p16 and p15 respectively,* show CN loss (CN = 0.8~ 1.32) in three samples of two patients. Previous research shows the inactivation of *CDKN2A* and *CDKN2B* are associated with tumorigenesis [35, 36]. 9p21 is a locus where frequent homozygous and heterozygous deletions occur in many primary tumors. *CDKN2A* and *CDKN2B* inhibit cyclin dependent kinase 4 (CDK4) and CDK6 and control cellular proliferation by preventing entry into the S phase of the cell cycle. Their inactivation may contribute to uncontrolled growth in cancer [35]. Young et al., in a cohort of 143 patients with primary invasive melanoma, showed that CNVs were common in melanoma and hemizygous or homozygous loss of *CDKN2A,* corresponding to 56% of cases [36]. There is no previous reporting on their involvement in gastric cancer prior to our work.

## Conclusions

To better understand the mechanism of occurrence and development of GAC, we selected 5 male patients with metastasis in lymph nodes, and performed exome sequencing in primary tumor tissues, matched normal tissues, and metastatic lymph nodes, followed by in-depth bioinformatics analysis and focused on the somatic mutations. We were able to confirm many known facts, and we have several discoveries:

1. We confirmed 30 candidate driver gene mutations in both primary and lymph nodes tissues, with another 7 candidate driver gene mutations only in primary tumors.
2. We identified the CNV of many genes previously known to drive GAC, including *EGFR* and *ERBB2*. Many of the genes in the amplified block may contribute to tumorigenesis synergistically, but mostly their impact is unknown today. Of special interest is a block within chromosome 17q12–21, where *ERBB2* is contained within. There are large-segment CN gains happened in17q12, 17q21.2 and 17q21.31, involving 14, 14, 63 genes respectively, with CN at 2.66~ 6.5 and in at least 3 distinct tissues. Through exome sequencing, we resolved the boundaries of these segmental amplifications to the level of genes.
3. Two genes, *CDKN2A* and *CDKN2B* show CN loss (CN = 0.8~ 1.32) in the primary tissues and metastatic lymph nodes of two patients. Previous research shows the inactivation of *CDKN2A* and *CDKN2B* are associated with tumorigenesis [35, 36].

Due to the relative small sample size, our discoveries in these categories should be considered as preliminary, at this current time. Nevertheless, our results indicate an important potential avenues for further work.

## Additional files

Additional file 1: **Table S1.** The complete list of all data with detailed information. (XLSX 12 kb)

Additional file 2: **Table S2.** Summary of all SNVs in primary tumor and metastasis tissues from 5 patients. (XLSX 307 kb)

Additional file 3: **Figure S1.** The pictures for the somatic mutations which were confirmed by Sanger sequencing and IGV (Integrative Genomics Viewer) software. (DOCX 6694 kb)

Additional file 4: **Table S3.** Somatic and germline mutations identified in primary tumor and metastasis tissues from 5 patients by Sanger sequencing and IGV. (XLSX 19 kb)

Additional file 5: **Table S4.** Summary of all CNVs from tumor and metastasis tissues of 5 patients. (XLSX 313 kb)

## Abbreviations

CDs: Coding sequences
CN: copy number
CNV: copy number variation
COSMIC: Catalogue Of Somatic Mutations In Cancer
GAC: gastric adenocarcinoma
GC: gastric cancer
NGS: next-generation sequencing
SNP: single nucleotide polymorphism
SNV: single nucleotide variant
VAF: variant allele frequency

## References

[1] Torre LA, Bray F, Siegel RL (2015). Global cancer statistics, 2012. CA Cancer J Clin.

[2] Lauren P (1965). The two histological main types of gastric carcinoma, an attempt at a histoclinical classification. Acta Pathol Microbiol Scand.

[3] Ferlay J, Soerjomataram I, Ervik M, et al. GLOBOCAN 2012 v1.0, Cancer incidence and mortality worldwide: IARC CancerBase No. 11: International Agency for Research on Cancer Web site; 2013. http://globocan.iarc.fr. Accessed 24 Nov 2014

[4] Chen W, Zheng R, Baade PD (2016). Cancer statistics in China, 2015. CA Cancer J Clin.

[5] Zheng L (2004). Molecular basis of gastric cancer development and progression. Gastric Cancer.

[6] Resende C (2011). Gastric cancer: basic aspects. Helicobacter.

[7] de Leon MP, de Leon MP (1994). Oncogenes and tumor suppressor genes. Familial and hereditary tumors. Springer Berlin Heidelberg.

[8] Li-Chang HH, Kasaian K, Ng Y (2015). Retrospective review using targeted deep sequencing reveals mutational differences between gastroesophageal junction and gastric carcinomas. BMC Cancer.

[9] Hu XT, He C (2013). Recent progress in the study of methylated tumor suppressor genes in gastric cancer. Chin J Cancer.

[10] Sato F, Meltzer SJ (2006). CpG island hypermethylation in progression of esophageal and gastric cancer. Cancer.

[11] Tamura G (2006). Alterations of tumor suppressor and tumor-related genes in the development and progression of gastric cancer. World J Gastroenterol.

[12] Guo M, Yan W, Verma M (2015). Epigenetics of gastric cancer. Cancer Epigenetics: Risk Assessment, Diagnosis, Treatment, and Prognosis.

[13] Wang K, Kan J, Yuen ST (2011). Exome sequencing identifies frequent mutation of ARID1A in molecular subtypes of gastric cancer. Nat Genet.

[14] Wang K, Yuen ST, Xu J (2014). Whole-genome sequencing and comprehensive molecular profiling identify new driver mutations in gastric cancer. Nat Genet.

[15] Guilford P, Hopkins J, Harraway J, McLeod M, McLeod N, Harawira P, Taite H, Scoular R, Miller A, Reeve AE (1998). E-cadherin germline mutations in familial gastric cancer. Nature.

[16] Miranda E, Destro A, Malesci A (2006). Genetic and epigenetic changes in primary metastatic and nonmetastatic colorectal cancer. Br J Cancer.

[17] Babraham Bioinformatics. http://www.bioinformatics.babraham.ac.uk/projects/fastqc/. 2106. Accessed 11 Nov 2016.

[18] USADELLAB.org. http://www.usadellab.org/cms/?page=trimmomatic. 2016. Accessed 11 Nov 2016.

[19] Li H, Durbin R (2009). Fast and accurate short read alignment with burrows-wheeler transform. Bioinformatics.

[20] McKenna A, Hanna M, Banks E, Sivachenko A, Cibulskis K, Kernytsky A, Garimella K, Altshuler D, Gabriel S, Daly M, DePristo MA. The Genome Analysis Toolkit: a MapReduce framework for analyzing next-generation. Genome Res. 2010;20(9):1297–1303. 10.1101/gr.107524.110 PMC2928508 20644199

[21] Cibulskis K, Lawrence MS, Carter SL, Sivachenko A, Jaffe D, Sougnez C, Gabriel S, Meyerson M, Lander ES, Getz G (2013). Sensitive detection of somatic point mutations in impure and heterogeneous cancer samples. Nat Biotechnol.

[22] Sherry ST, Ward MH, Kholodov M (2001). dbSNP: the NCBI database of genetic variation. Nucleic Acids Res.

[23] D'Aurizio R, Pippucci T, Tattini L (2016). Enhanced copy number variants detection from whole-exome sequencing data using EXCAVATOR2. Nucleic Acids Res.

[24] Thorvaldsdóttír H (2013). Integrated genomics viewer (IGV): high-performance genomics data visualization and exploration. Brief Bioinform.

[25] Exome Aggregation Consortium (ExAC). 2016. http://exac.broadinstitute.org. Accessed 21 Oct 2016.

[26] Liu H, Li F, Zhu Y (2016). Whole-exome sequencing to identify somatic mutations in peritoneal metastatic gastric adenocarcinoma: a preliminary study. Oncotarget.

[27] Chen K, Yang D, Li X (2015). Mutational landscape of gastric adenocarcinoma in Chinese: implications for prognosis and therapy. Proc Natl Acad Sci U S A.

[28] Zang ZJ, Cutcutache I, Poon SL (2012). Exome sequencing of gastric adenocarcinoma identifies recurrent somatic mutations in cell adhesion and chromatin remodeling genes. Nat Genet.

[29] Cancer-geneticsweb. http://www.cancer-genetics.org/genes_a.htm. 2016. Accessed 21 Oct 2016.

[30] Catalogue Of Somatic Mutations In Cancer. 2016. http://cancer.sanger.ac.uk/census. Accessed 11 Nov 2016.

[31] Liang L, Fang JY, Xu J (2016). Gastric cancer and gene copy number variation: emerging cancer drivers for targeted therapy. Oncogene.

[32] Katoh M, Katoh M (2004). Evolutionary recombination hotspot around GSDML-GSDM locus is closely linked to the oncogenomic recombination hotspot around the PPP1R1B-ERBB2-GRB7 amplicon. Int J Oncol.

[33] Maqani N, Belkhiri A, Moskaluk C (2006). Molecular dissection of 17q12 amplicon in upper gastrointestinal adenocarcinomas. Mol Cancer Res.

[34] Varis A, Wolf M, Monni O (2002). Targets of gene amplification and overexpression at 17q in gastric cancer. Cancer Res.

[35] Suzuki H, Zhou X, Yin J (1995). Intragenic mutations of CDKN2B and CDKN2A in primary human esophageal cancers. Hum Mol Genet.

[36] Young RJ, Waldeck K, Martin C (2014). Loss of CDKN2A expression is a frequent event in primary invasive melanoma and correlates with sensitivity to the CDK4/6 inhibitor PD0332991 in melanoma cell lines. Pigment Cell Melanoma Res.

