# Supplementary material for: Genomic alterations in gastric cancers discovered via whole-exome sequencing
Source: BMC Cancer. 2018 Dec 19;18:1270. doi: 10.1186/s12885-018-5097-8 (PMC6299976; doi:10.1186/s12885-018-5097-8)
Supplement: Supplementary file 3 — Figure S1. The pictures for the somatic mutations which were confirmed by Sanger sequencing and IGV (Integrative Genomics Viewer) software. (DOCX 6694 kb) [file 12885_2018_5097_MOESM3_ESM.docx]

**Figure S1.** **Thirty-seven somatic mutations were confirmed by using Integrative Genomics Viewer (IGV) and Sanger sequencing.** P1~P5 are Patient IDs; letter L indicates lymph node metastatic tissue; and S primary tumor tissue. Red means mutation samples.

**
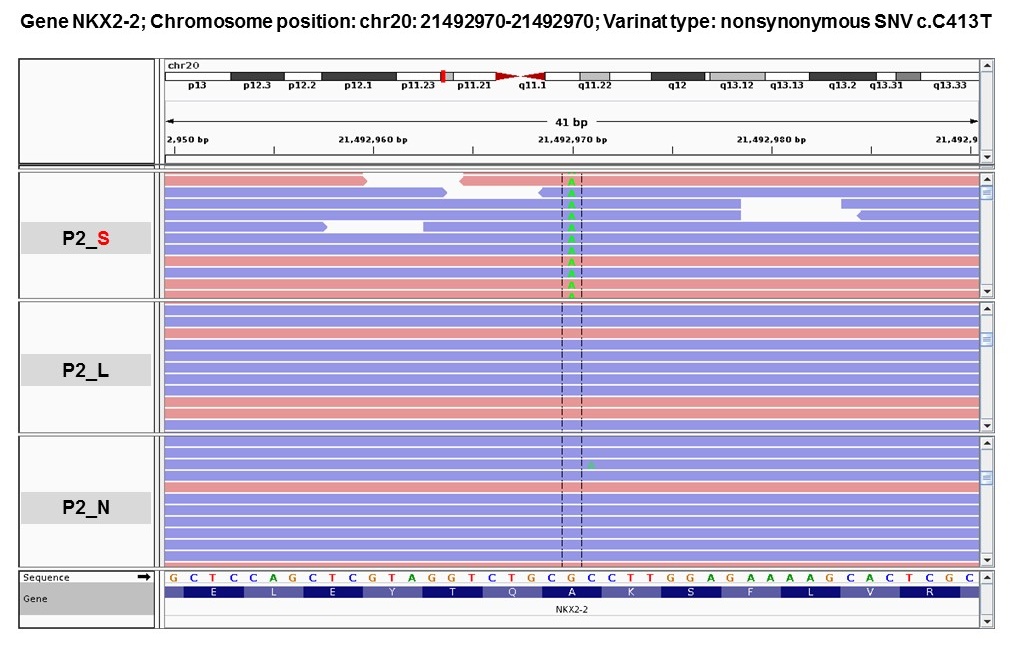

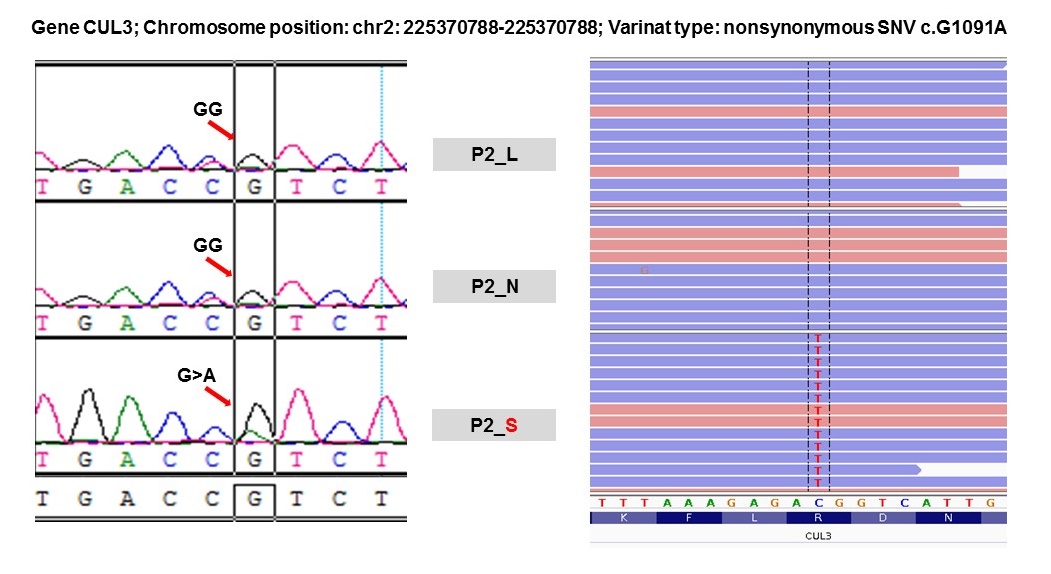
**

**
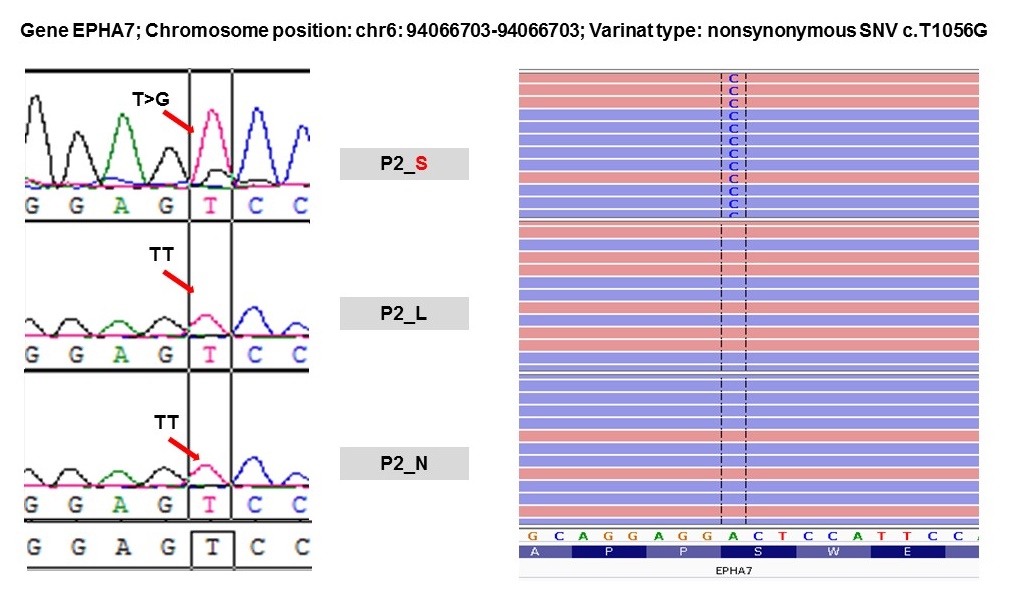

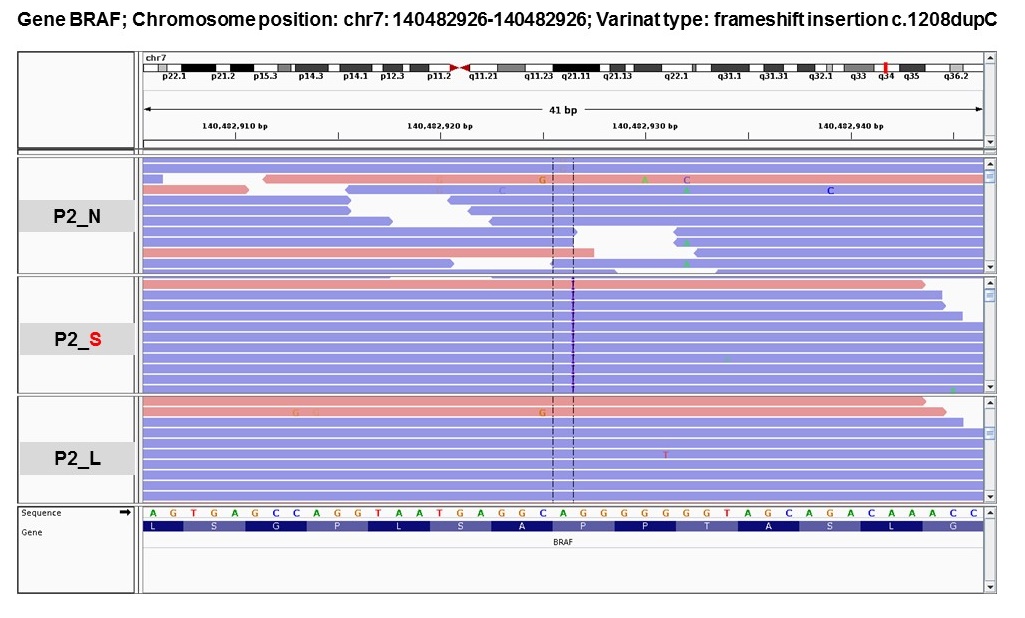

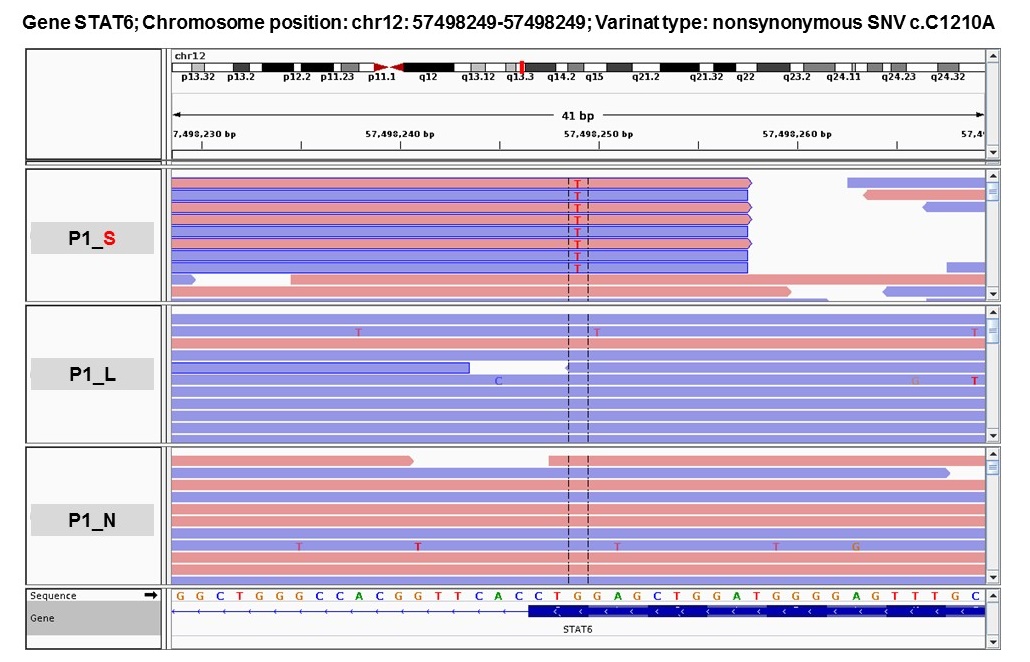

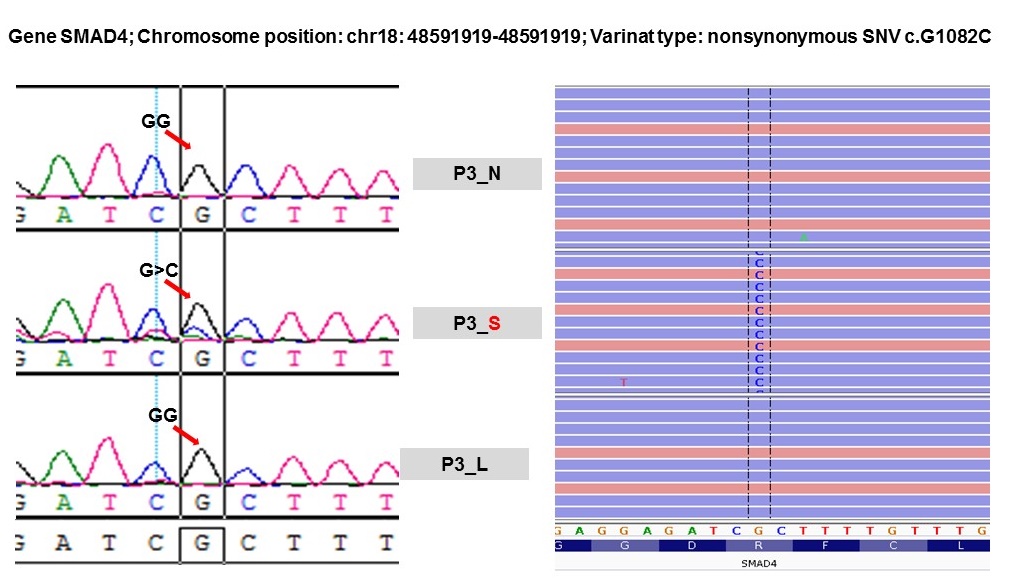

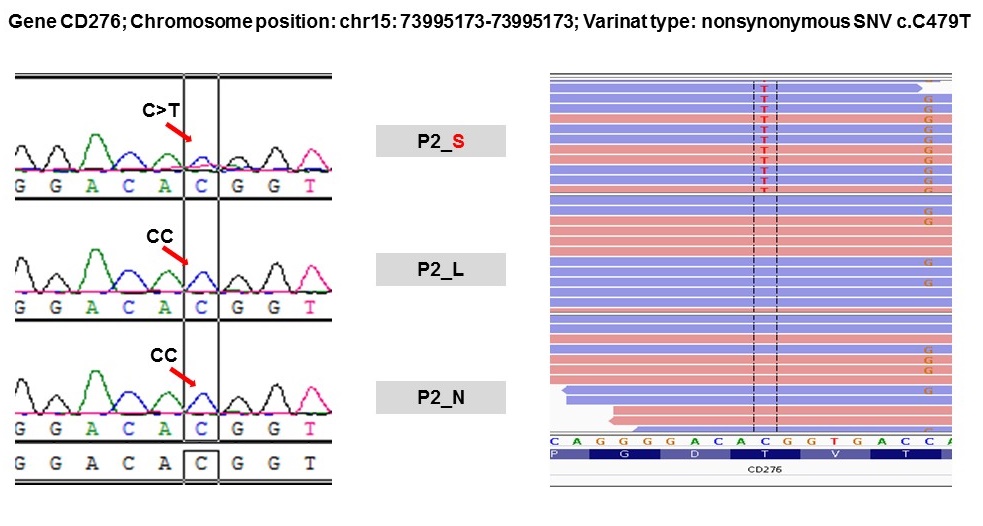

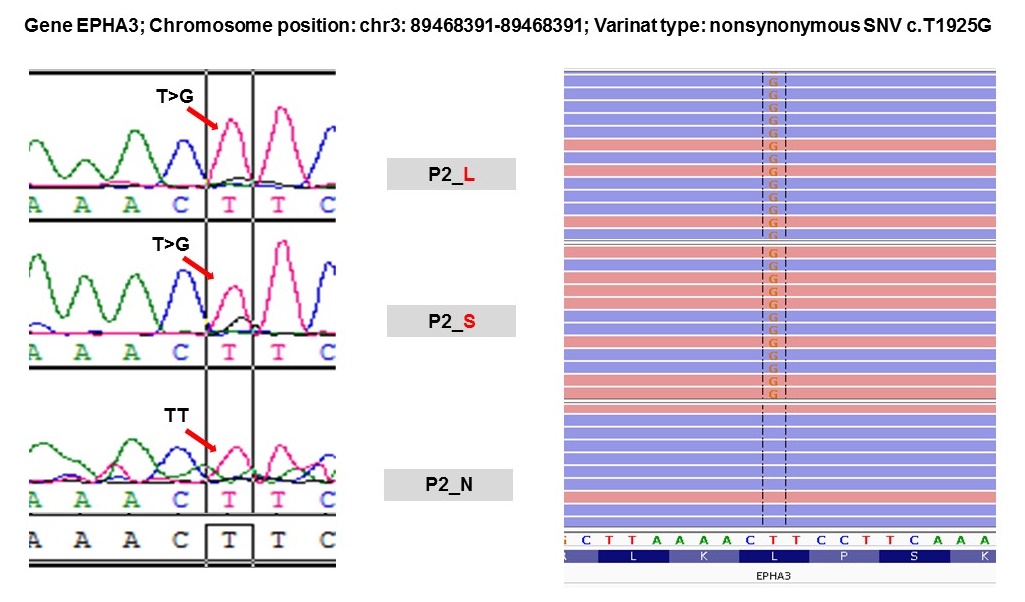

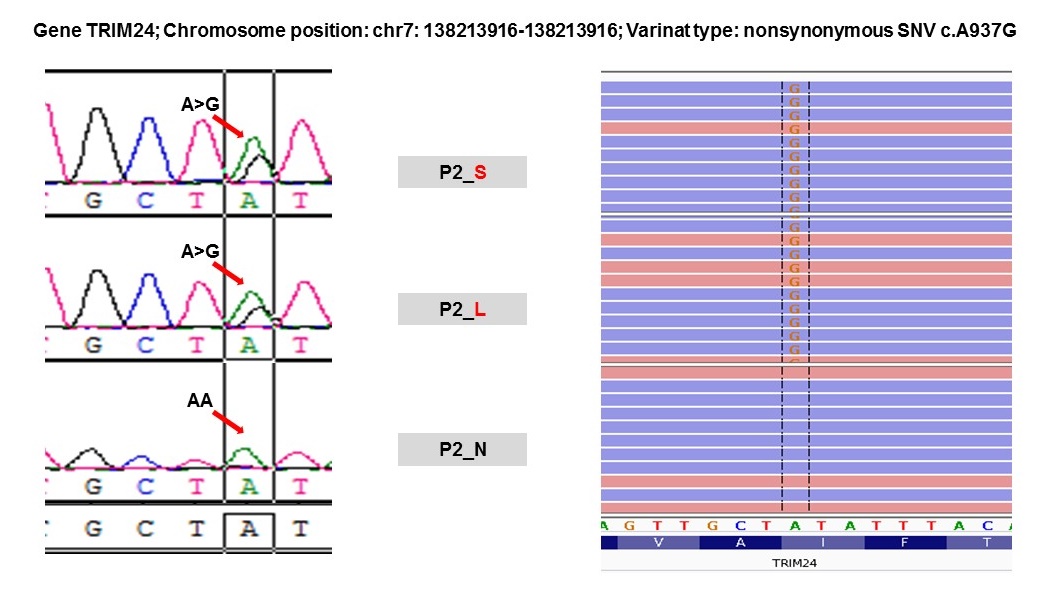

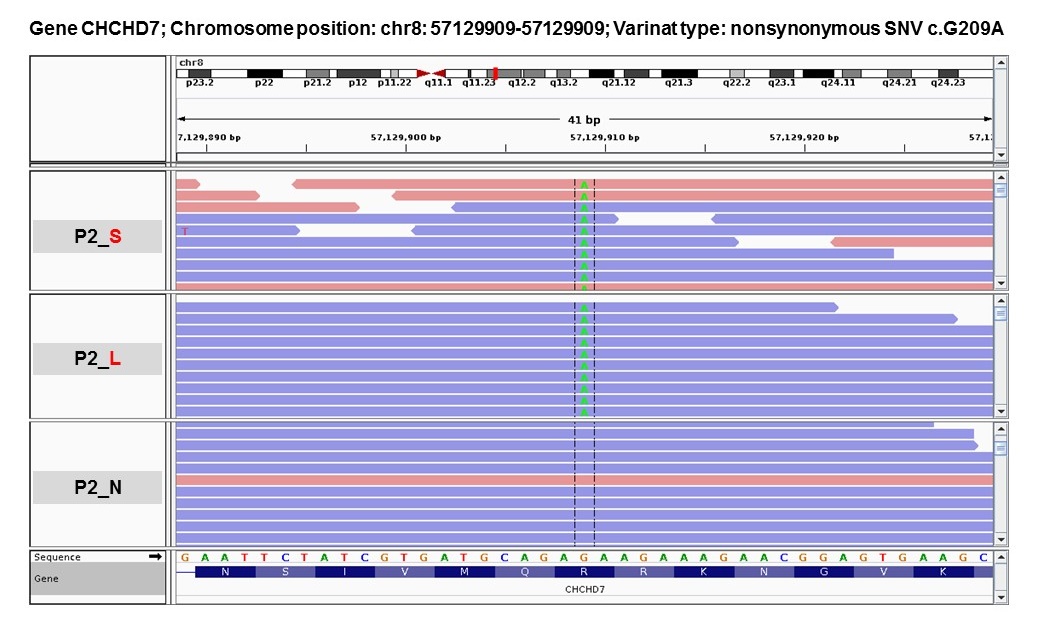

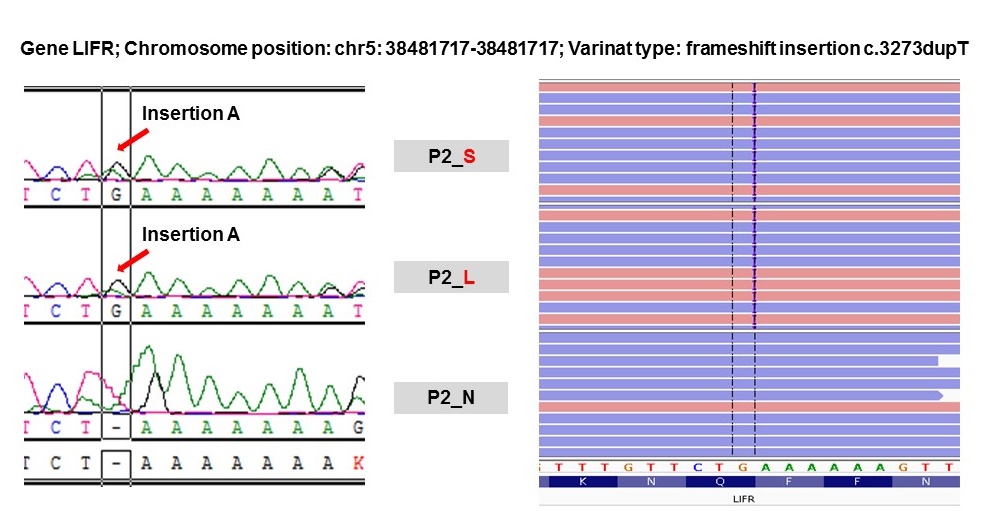

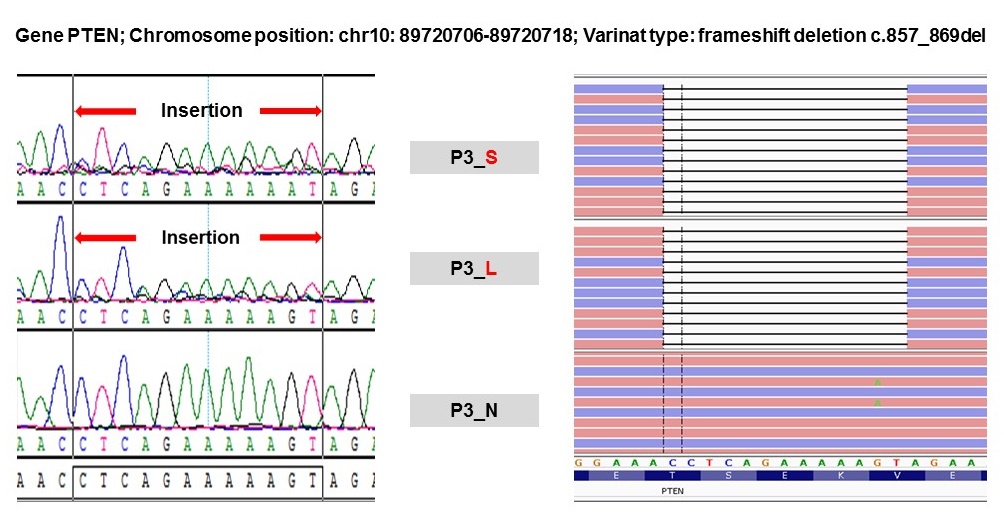

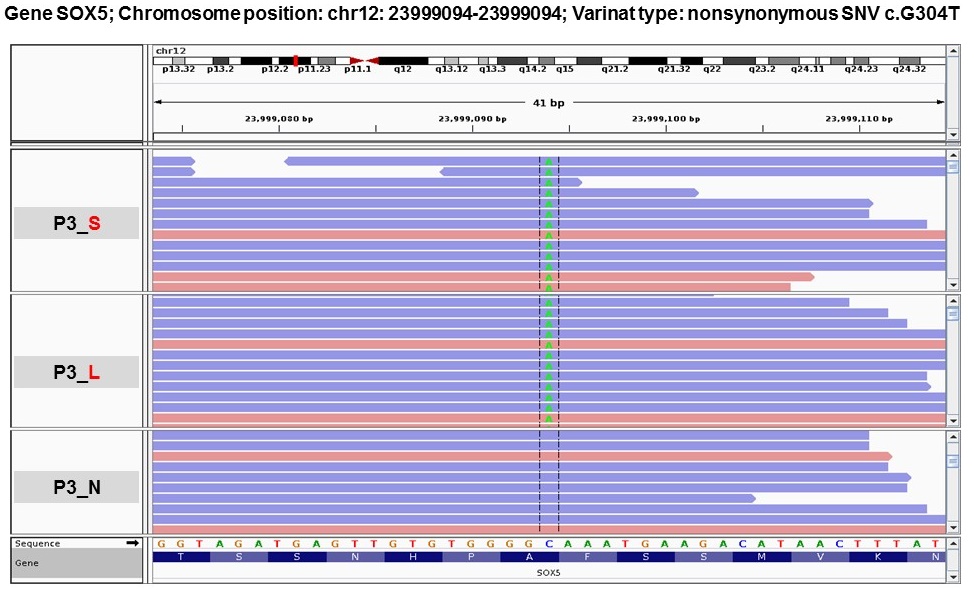

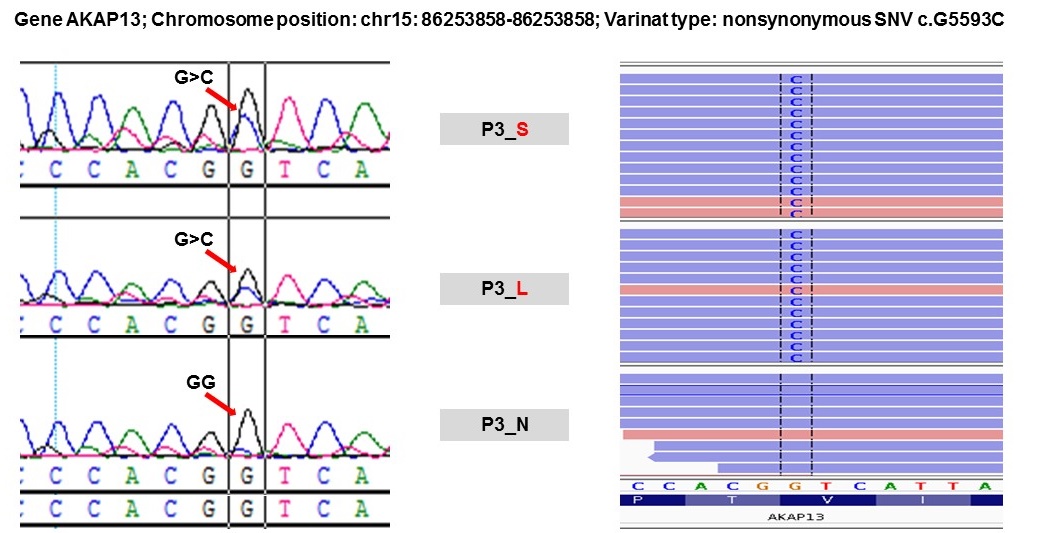

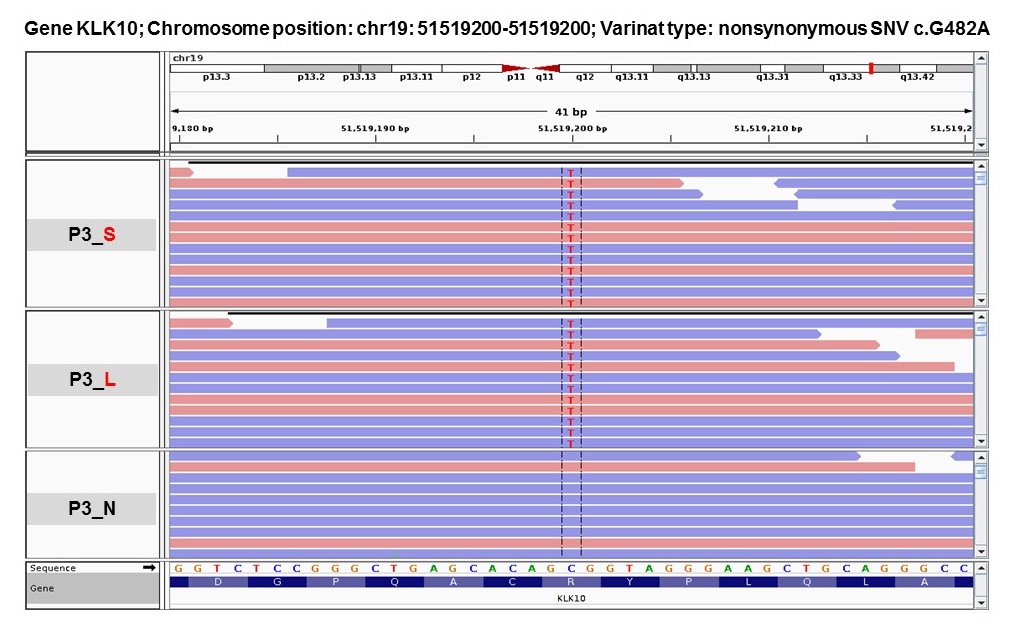

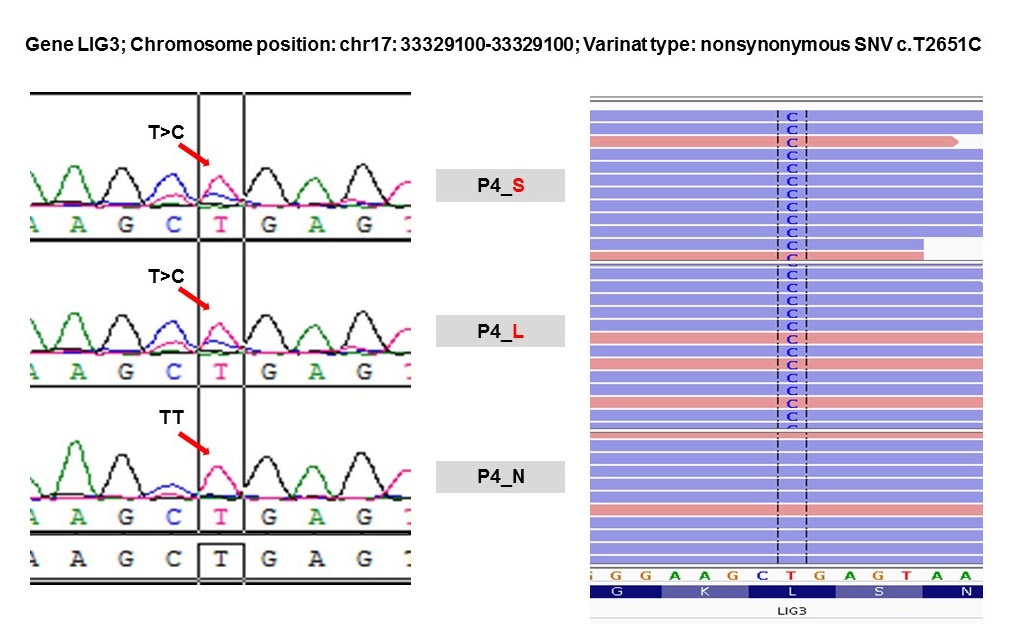

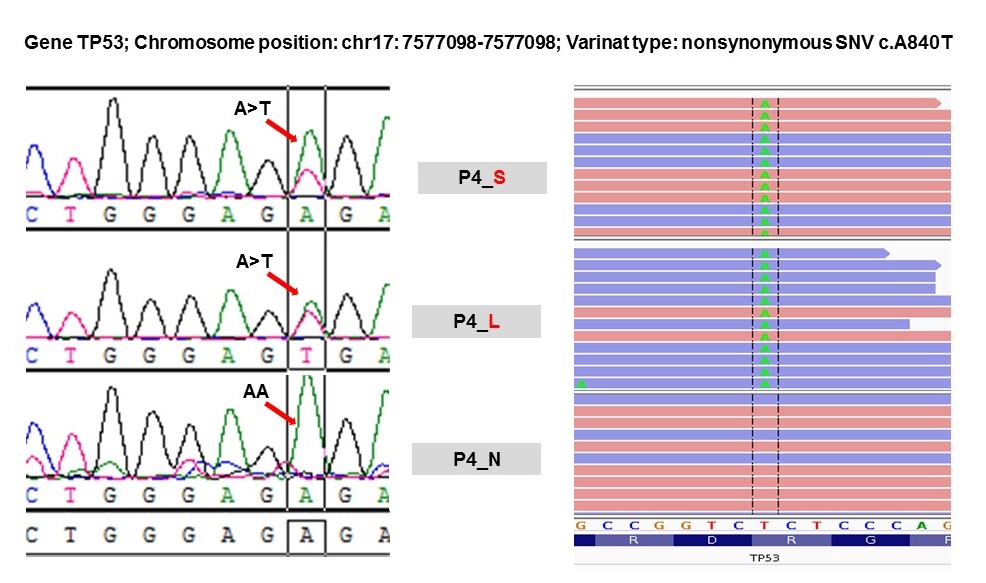
**

**
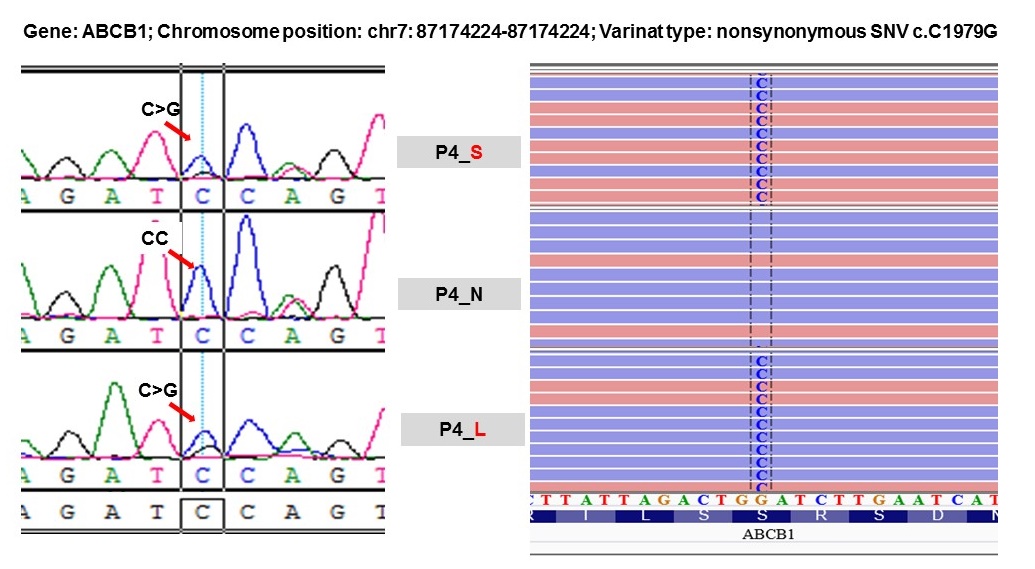

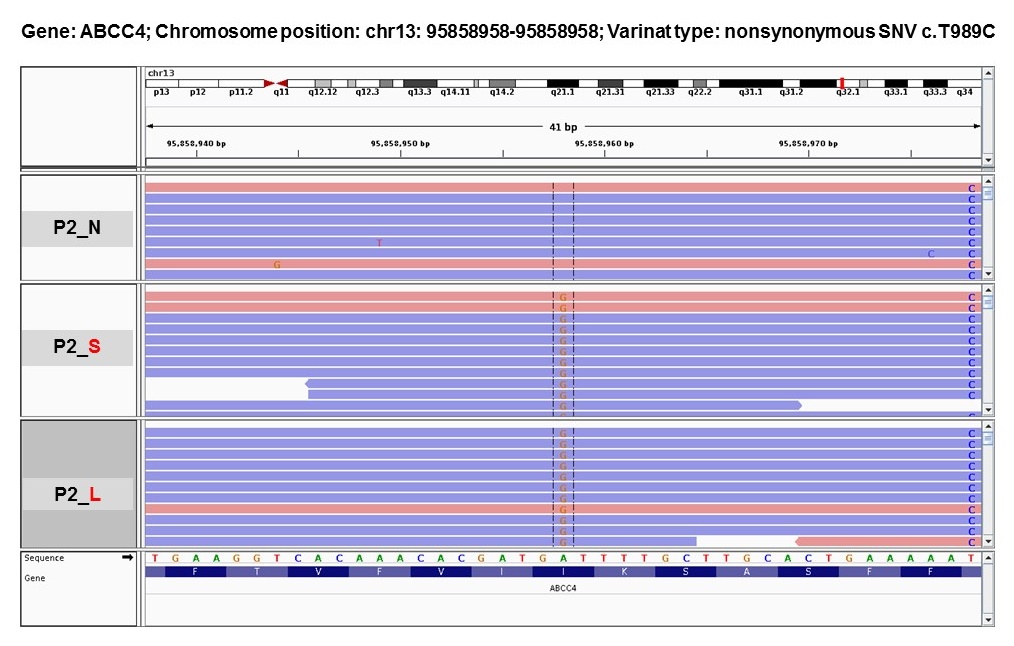

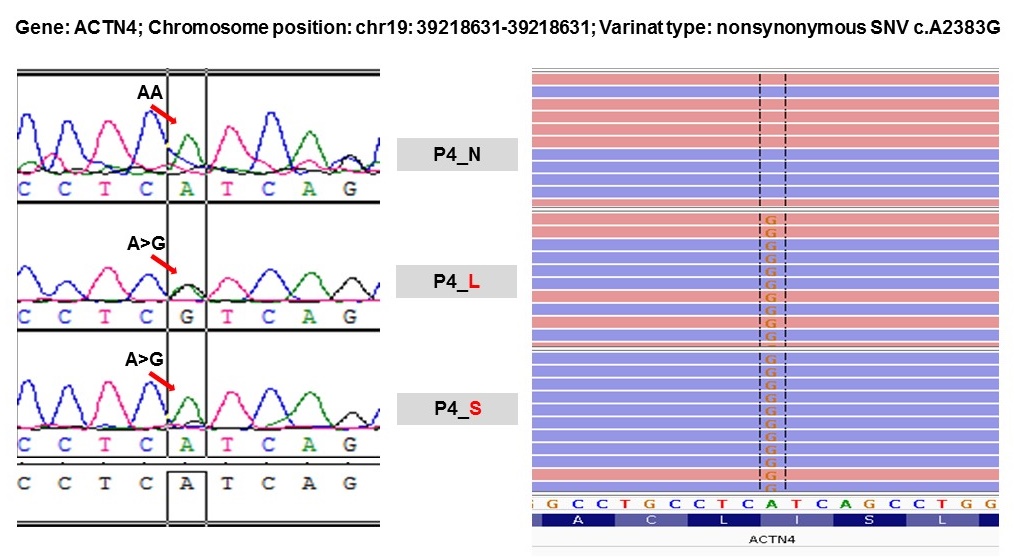

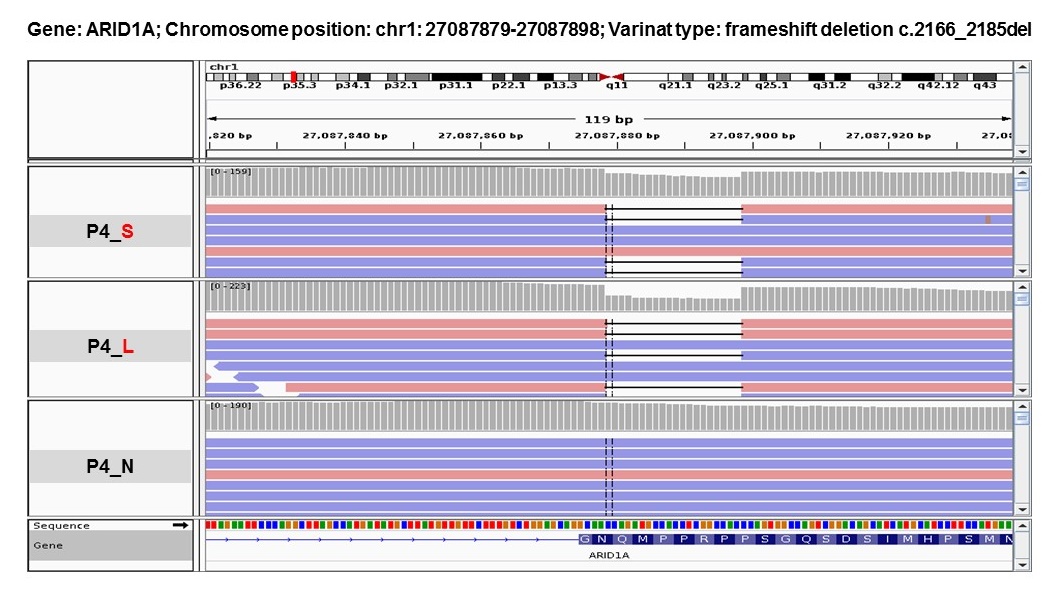

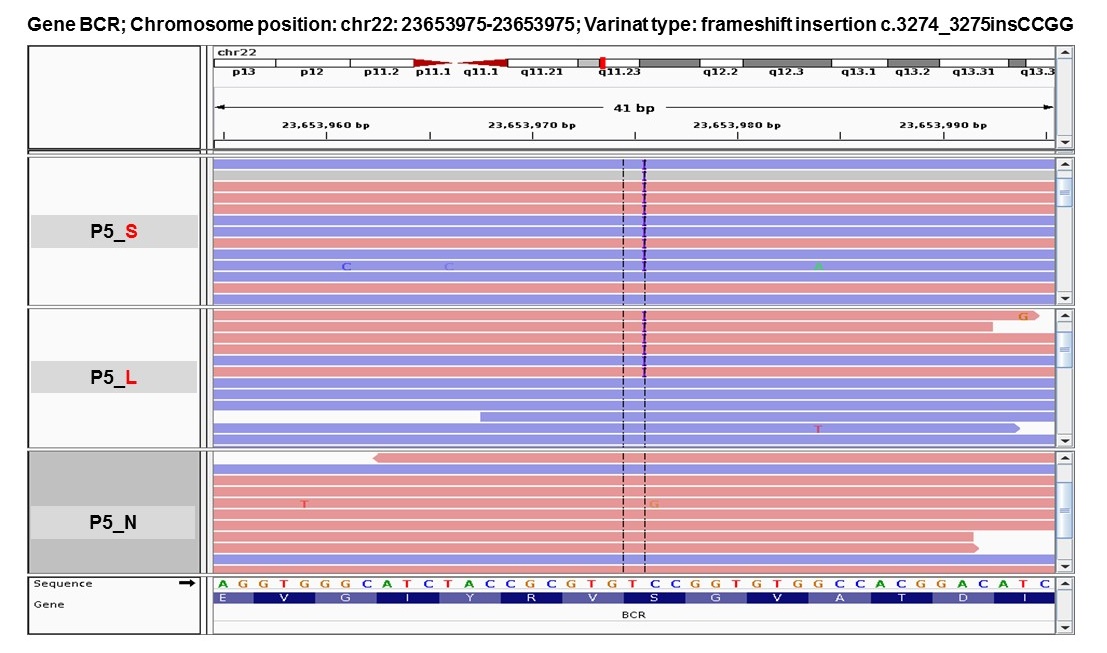

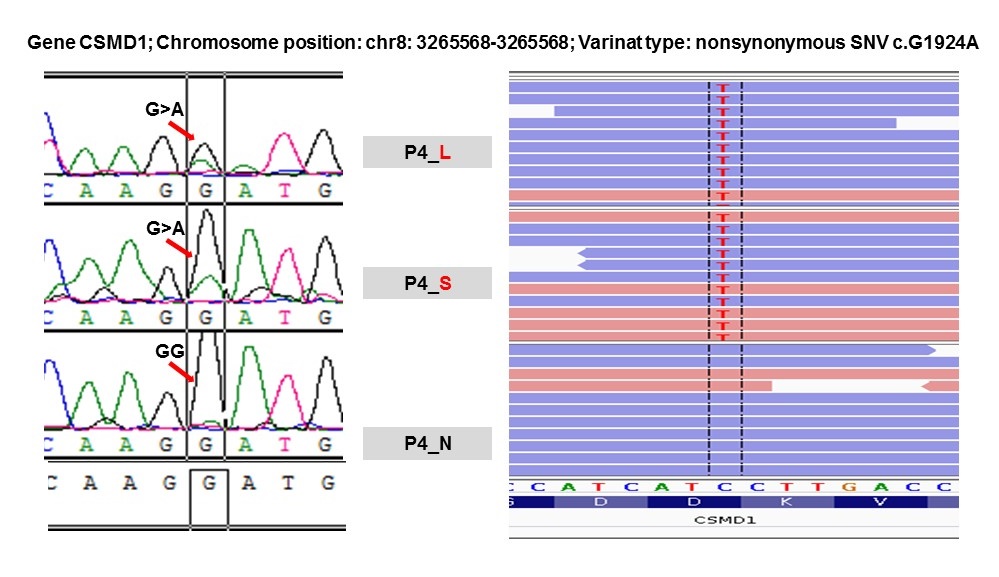

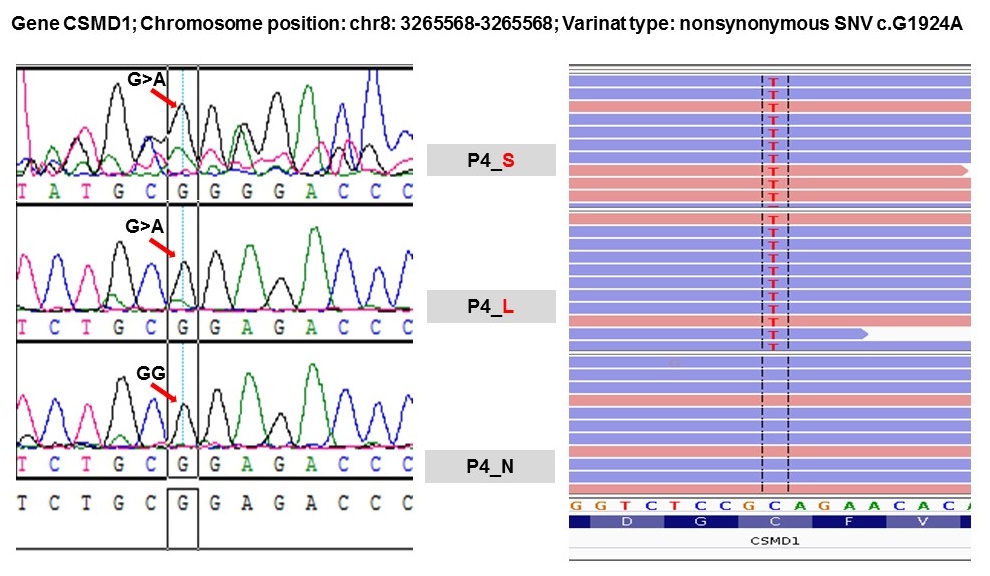

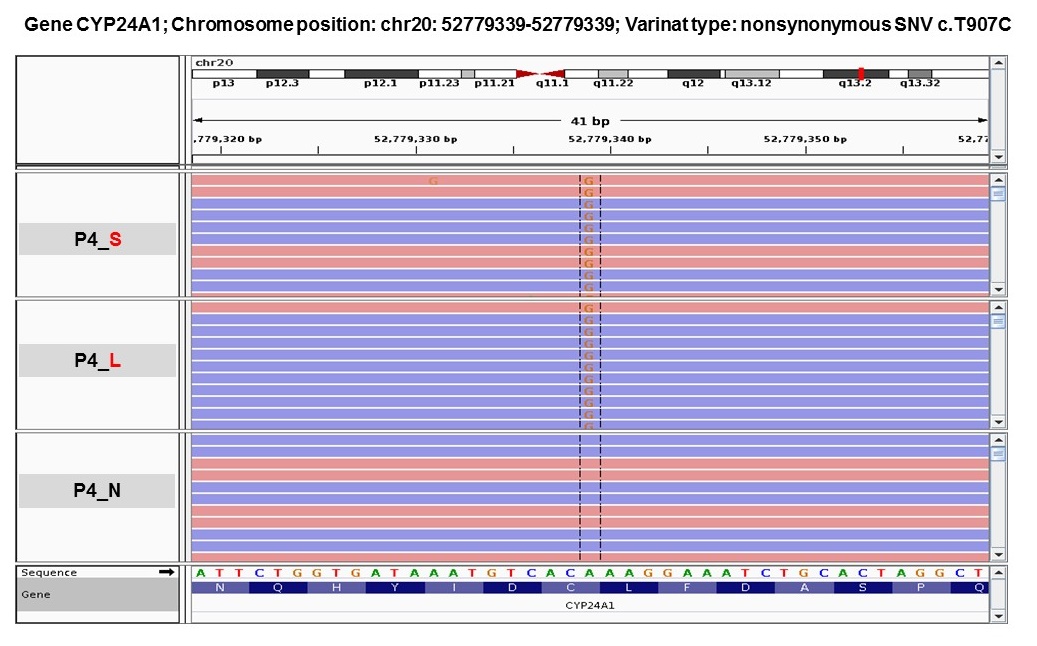

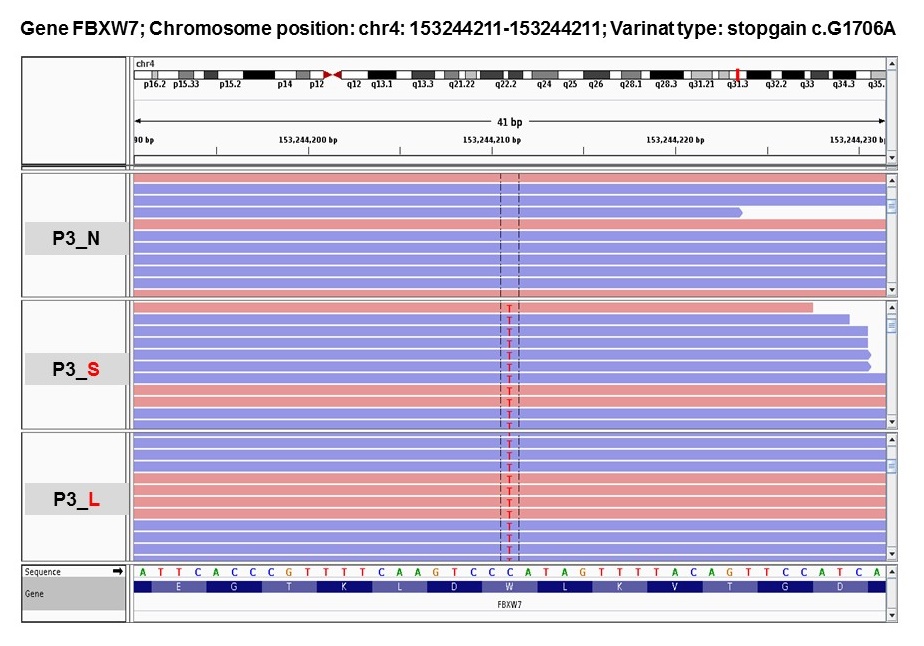

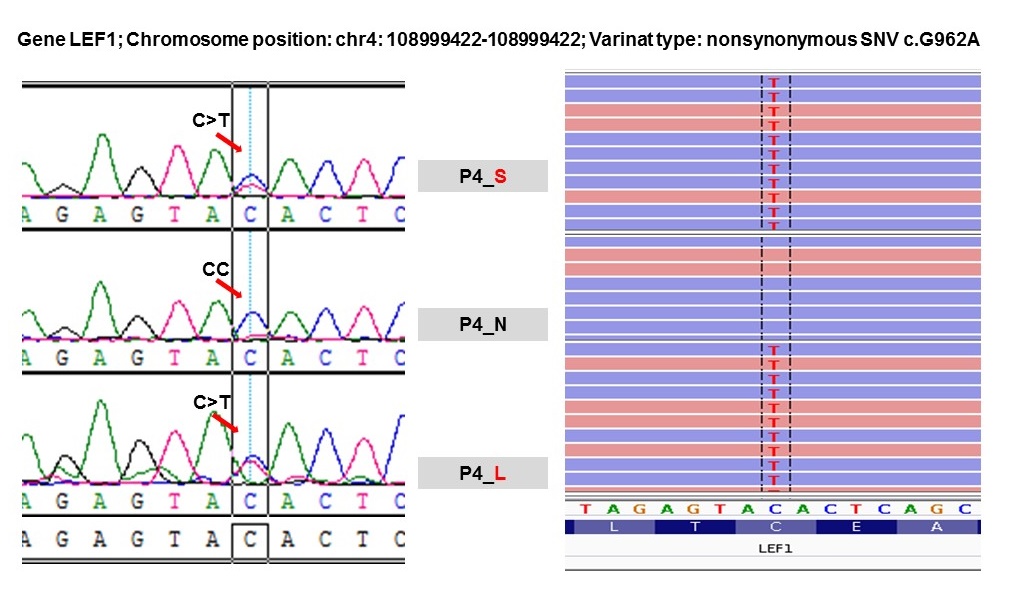

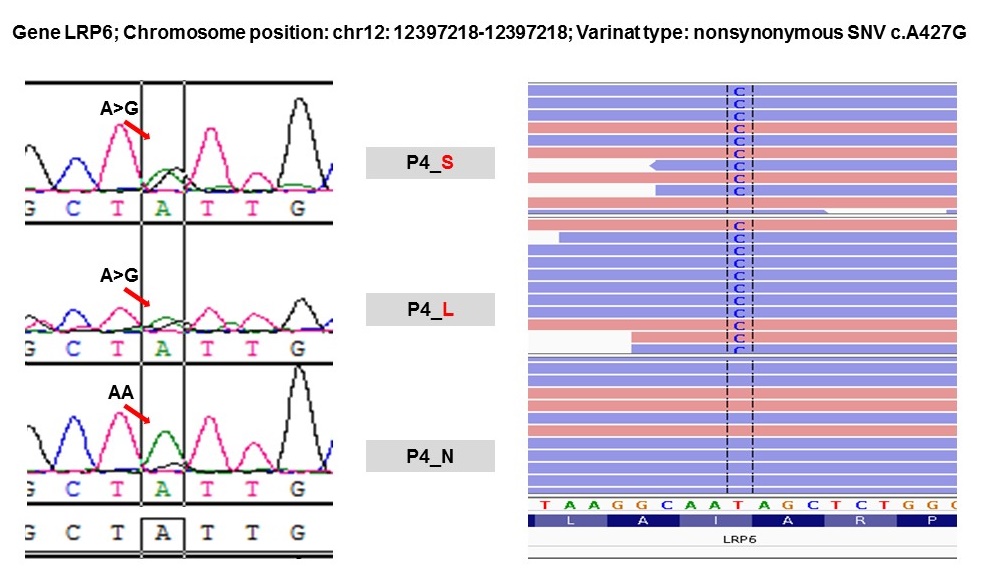

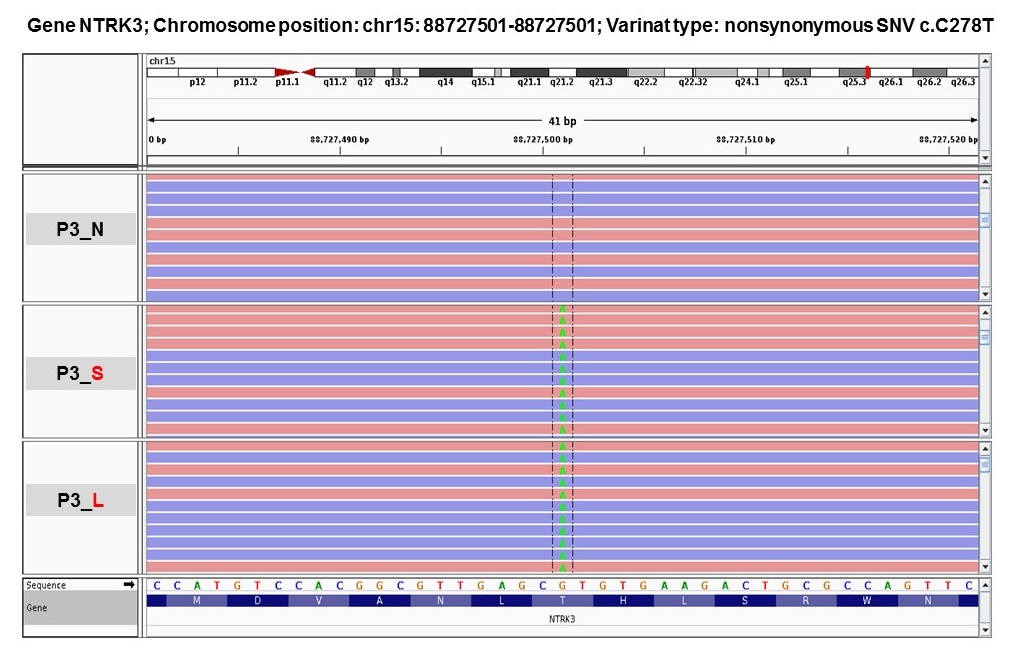

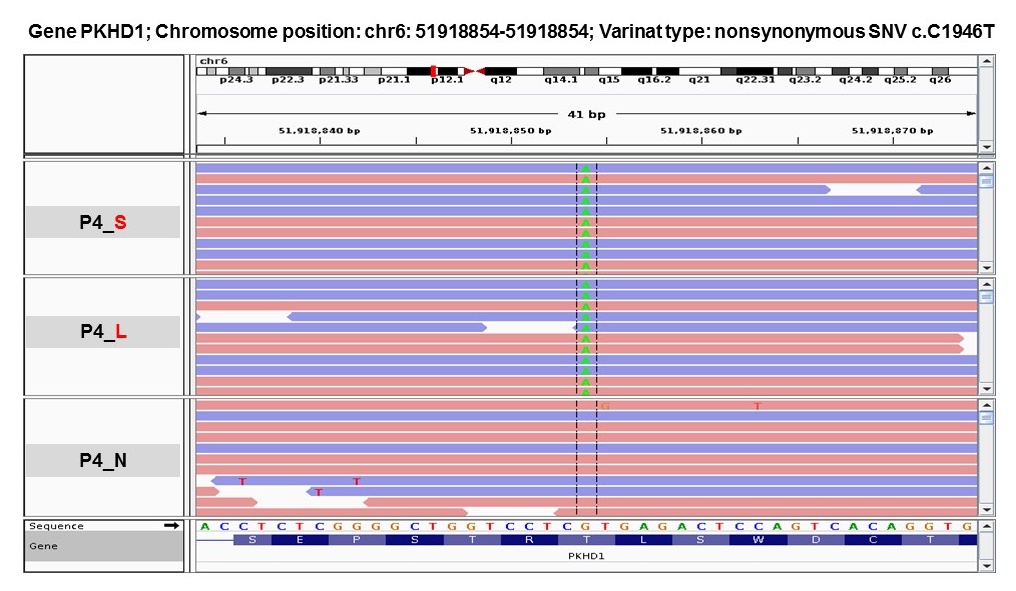

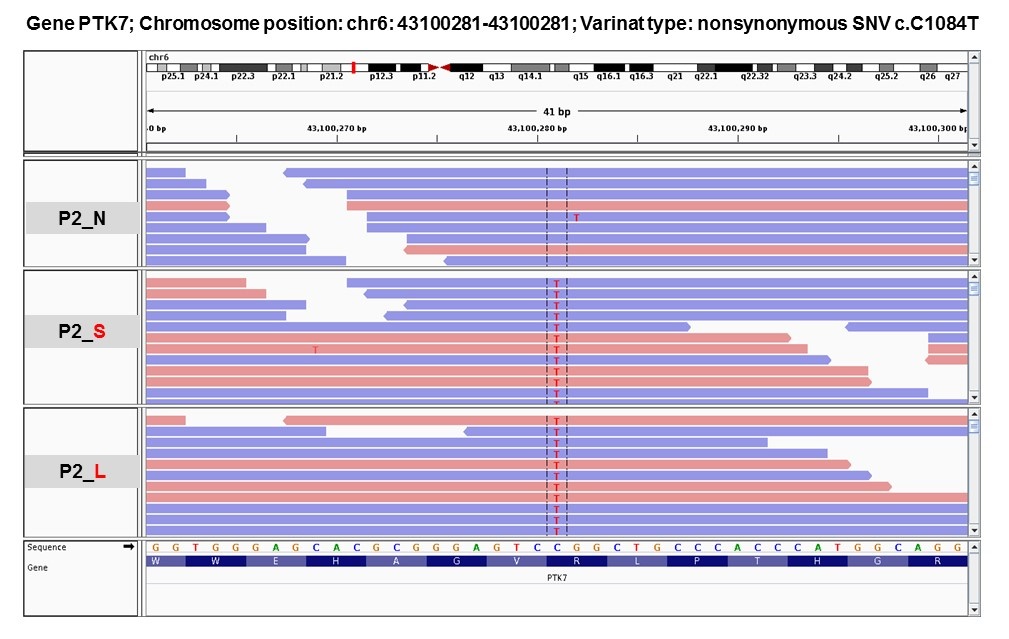

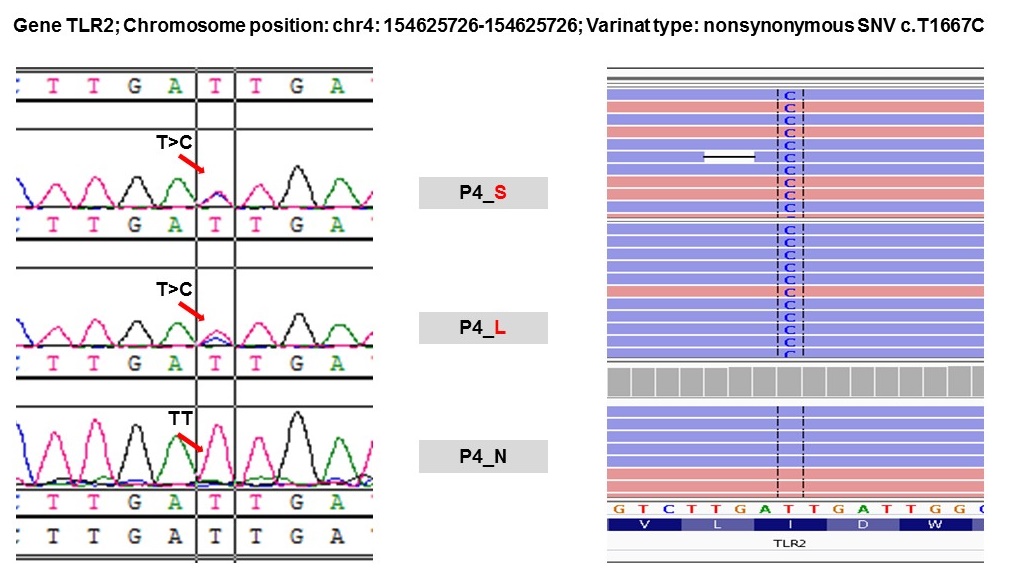

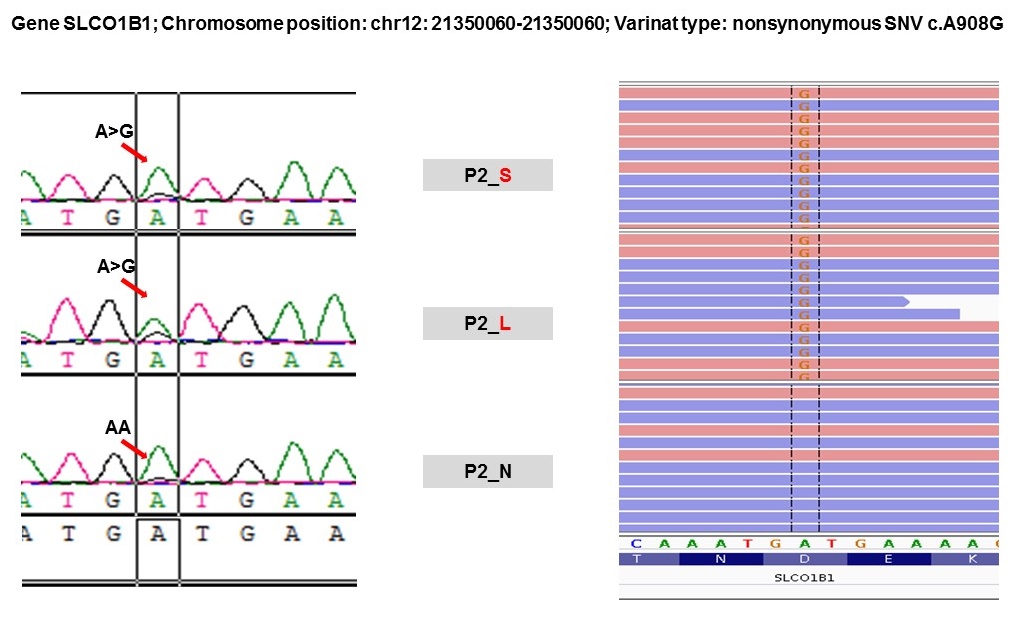

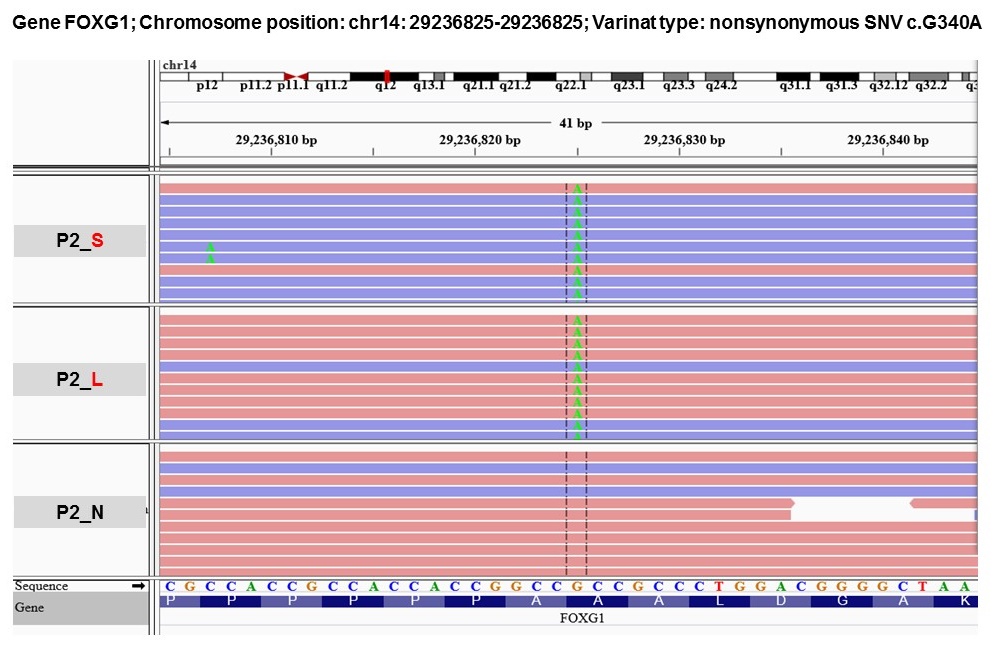

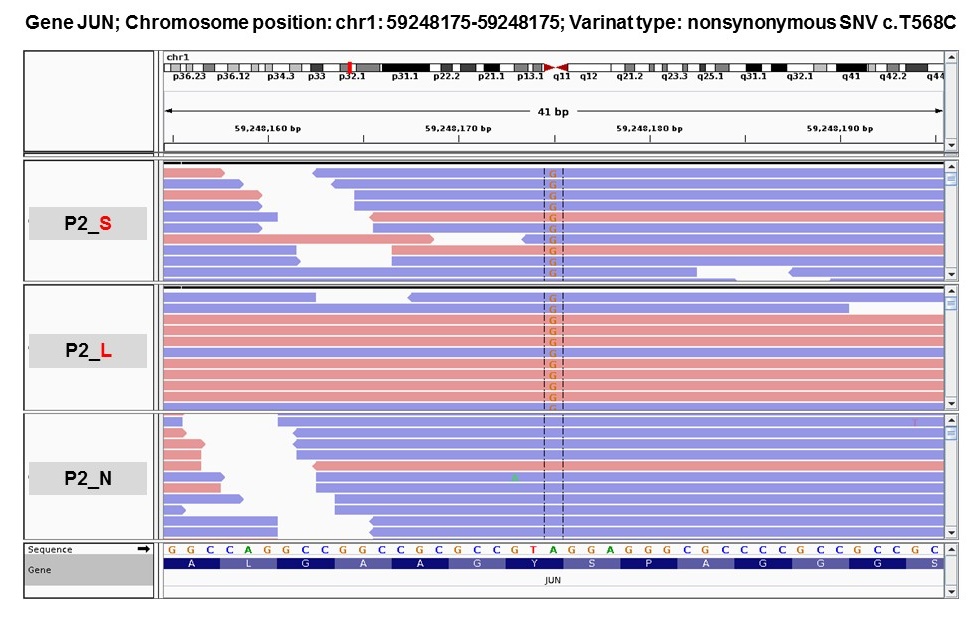

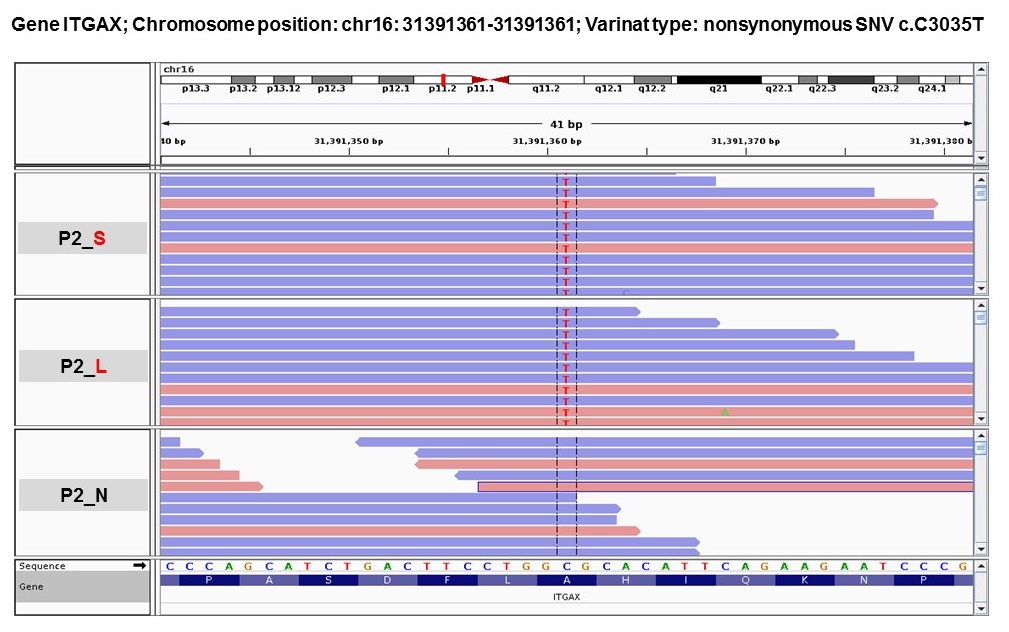

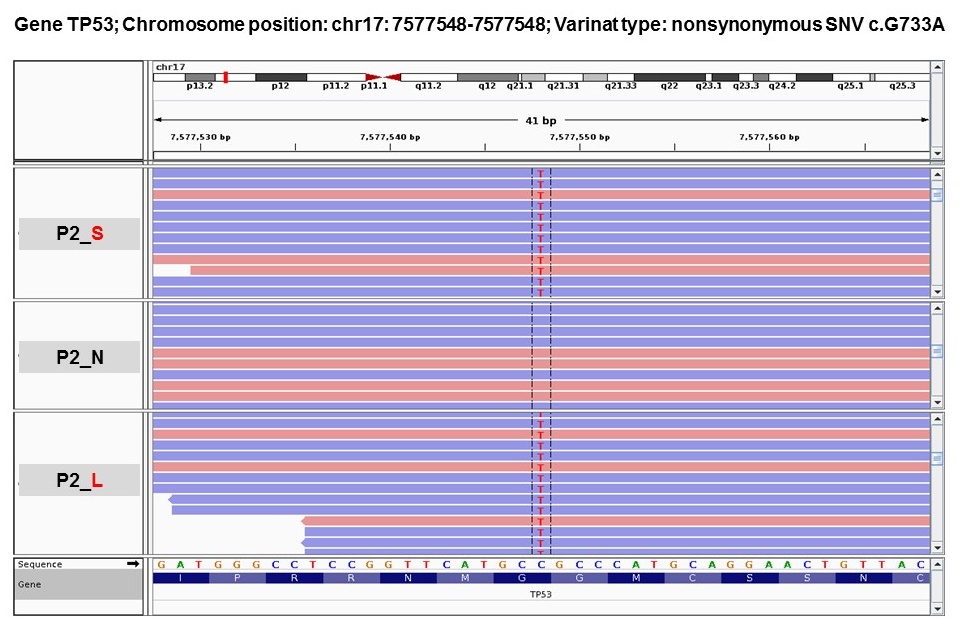
**
